# Machine learning for diagnosis of myocardial infarction using cardiac troponin concentrations

**DOI:** 10.1038/s41591-023-02325-4

**Published:** 2023-05-11

**Authors:** Dimitrios Doudesis, Kuan Ken Lee, Jasper Boeddinghaus, Anda Bularga, Amy V. Ferry, Chris Tuck, Matthew T. H. Lowry, Pedro Lopez-Ayala, Thomas Nestelberger, Luca Koechlin, Miguel O. Bernabeu, Lis Neubeck, Atul Anand, Karen Schulz, Fred S. Apple, William Parsonage, Jaimi H. Greenslade, Louise Cullen, John W. Pickering, Martin P. Than, Alasdair Gray, Christian Mueller, Nicholas L. Mills, CoDE-ACS Investigators, A. Mark Richards, Chris Pemberton, Richard W. Troughton, Sally J. Aldous, Anthony F. T. Brown, Emily Dalton, Chris Hammett, Tracey Hawkins, Shanen O’Kane, Kate Parke, Kimberley Ryan, Jessica Schluter, Karin Wild, Desiree Wussler, Òscar Miró, F. Javier Martin-Sanchez, Dagmar I. Keller, Michael Christ, Andreas Buser, Maria Rubini Giménez, Stephanie Barker, Jennifer Blades, Andrew R. Chapman, Takeshi Fujisawa, Dorien M. Kimenai, Jeremy Leung, Ziwen Li, Michael McDermott, David E. Newby, Stacey D. Schulberg, Anoop S. V. Shah, Andrew Sorbie, Grace Soutar, Fiona E. Strachan, Caelan Taggart, Daniel Perez Vicencio, Yiqing Wang, Ryan Wereski, Kelly Williams, Christopher J. Weir, Colin Berry, Alan Reid, Donogh Maguire, Paul O. Collinson, Yader Sandoval, Stephen W. Smith

**Affiliations:** 1 British Heart Foundation/University Centre for Cardiovascular Science, University of Edinburgh, Edinburgh, UK; 2 Usher Institute, University of Edinburgh, Edinburgh, UK; 3 Cardiovascular Research Institute Basel and Department of Cardiology, University Hospital Basel, University of Basel, Basel, Switzerland; 4 Department of Cardiac Surgery, University Hospital Basel, University of Basel, Basel, Switzerland; 5 The Bayes Centre, The University of Edinburgh, Edinburgh, UK; 6 School of Health and Social Care, Edinburgh Napier University, Edinburgh, UK; 7 Cardiac Biomarkers Trials Laboratory, Hennepin Healthcare Research Institute, Minneapolis, MN USA; 8 Departments of Laboratory Medicine and Pathology, Hennepin County Medical Center and University of Minnesota, Minneapolis, MN USA; 9 Australian Centre for Health Service Innovation, Centre for Healthcare Transformation, Queensland University of Technology, Brisbane, Queensland Australia; 10 Emergency and Trauma Centre, Royal Brisbane and Women’s Hospital, Brisbane, Queensland Australia; 11 School of Medicine, University of Queensland, Brisbane, Queensland Australia; 12 Faculty of Health, Queensland University of Technology, Brisbane, Queensland Australia; 13 Department of Medicine, University of Otago, Christchurch, New Zealand; 14 Emergency Department, Christchurch Hospital, Christchurch, New Zealand; 15 Emergency Medicine Research Group Edinburgh, Royal Infirmary of Edinburgh, Edinburgh, UK; 16 Christchurch Heart Institute, Department of Medicine, University of Otago, Christchurch, New Zealand; 17 Cardiovascular Research Institute, National University of Singapore, Singapore, Singapore; 18 Cardiology Department, Christchurch Hospital, Christchurch, New Zealand; 19 Department of Cardiology, University Hospital Basel, University of Basel, Basel, Switzerland; 20 Emergency Department, Hospital Clinic, Barcelona, Spain; 21 Servicio de Urgencias, Hospital Clínico San Carlos, Madrid, Spain; 22 Emergency Department, University Hospital Zurich, Zurich, Switzerland; 23 Emergency Department, Kantonsspital Luzern, Luzern, Switzerland; 24 Department of Hematology and Blood Bank, University Hospital Basel, Basel, Switzerland; 25 Department of Cardiology, Heart Center Leipzig, University of Leipzig, Leipzig, Germany; 26 Institute of Cardiovascular and Medical Sciences, University of Glasgow, Glasgow, UK; 27 Department of Biochemistry, Queen Elizabeth University Hospital, Glasgow, UK; 28 Emergency Medicine Department, Glasgow Royal Infirmary, Glasgow, UK; 29 Departments of Clinical Blood Sciences and Cardiology, St. George’s University Hospitals National Health Service Trust and St. George’s University of London, London, UK; 30 Minneapolis Heart Institute, Minneapolis Heart Institute Foundation, Minneapolis, MN USA; 31 Department of Emergency Medicine, Hennepin County Medical Center and University of Minnesota, Minneapolis, MN USA

**Keywords:** Diagnostic markers, Myocardial infarction

## Abstract

Although guidelines recommend fixed cardiac troponin thresholds for the diagnosis of myocardial infarction, troponin concentrations are influenced by age, sex, comorbidities and time from symptom onset. To improve diagnosis, we developed machine learning models that integrate cardiac troponin concentrations at presentation or on serial testing with clinical features and compute the Collaboration for the Diagnosis and Evaluation of Acute Coronary Syndrome (CoDE-ACS) score (0–100) that corresponds to an individual’s probability of myocardial infarction. The models were trained on data from 10,038 patients (48% women), and their performance was externally validated using data from 10,286 patients (35% women) from seven cohorts. CoDE-ACS had excellent discrimination for myocardial infarction (area under curve, 0.953; 95% confidence interval, 0.947–0.958), performed well across subgroups and identified more patients at presentation as low probability of having myocardial infarction than fixed cardiac troponin thresholds (61 versus 27%) with a similar negative predictive value and fewer as high probability of having myocardial infarction (10 versus 16%) with a greater positive predictive value. Patients identified as having a low probability of myocardial infarction had a lower rate of cardiac death than those with intermediate or high probability 30 days (0.1 versus 0.5 and 1.8%) and 1 year (0.3 versus 2.8 and 4.2%; *P* < 0.001 for both) from patient presentation. CoDE-ACS used as a clinical decision support system has the potential to reduce hospital admissions and have major benefits for patients and health care providers.

## Abstract (web summary)

A clinical decision support system for diagnosis of myocardial infarction, based on machine learning models that use a single measurement of high-sensitivity troponin, outperforms clinical guidelines that use fixed cardiac troponin thresholds for diagnosis.

## Main

High-sensitivity cardiac troponin assays have enabled the adoption of accelerated diagnostic pathways for the assessment of patients with symptoms suggestive of acute myocardial infarction^1–10^. These pathways are now recommended by national and international clinical practice guidelines, but they have some important limitations^11–13^. First, they use fixed troponin thresholds for all patients, which do not account for age, sex or comorbidities that are known to influence cardiac troponin concentrations^5,14–17^. Second, they are based on specific time points for serial testing, which can be challenging to apply consistently in busy emergency departments^18^. Third, they categorize patients as low, intermediate or high risk of myocardial infarction based on troponin thresholds alone and do not consider other important information, such as the time of symptom onset or findings on the electrocardiogram^19^. Finally, although these pathways perform well to rule out myocardial infarction, identifying those with the condition is more challenging, and the performance of the 99th percentile diagnostic threshold is inconsistent in men and women, in older patients and in those with comorbidities^20–24^. In this study, we hypothesized that machine learning approaches to integrate cardiac troponin as a continuous measure and clinical features known to influence concentrations may provide a more individualized approach to assess probability and improve the diagnosis of myocardial infarction.

In a prespecified analysis of the High-Sensitivity Troponin in the Evaluation of Patients with Suspected Acute Coronary Syndrome (High-STEACS) trial^25^, we evaluated the diagnostic performance of guideline-recommended cardiac troponin thresholds and developed a clinical decision support system called the Collaboration for the Diagnosis and Evaluation of Acute Coronary Syndrome (CoDE-ACS) that uses machine learning models to calculate the probability of myocardial infarction for an individual patient. We then externally validated the diagnostic performance of CoDE-ACS and compared performance with guideline-recommended pathways to demonstrate how it could be used in clinical practice.

## Results

**Extended Data Fig. 1:**
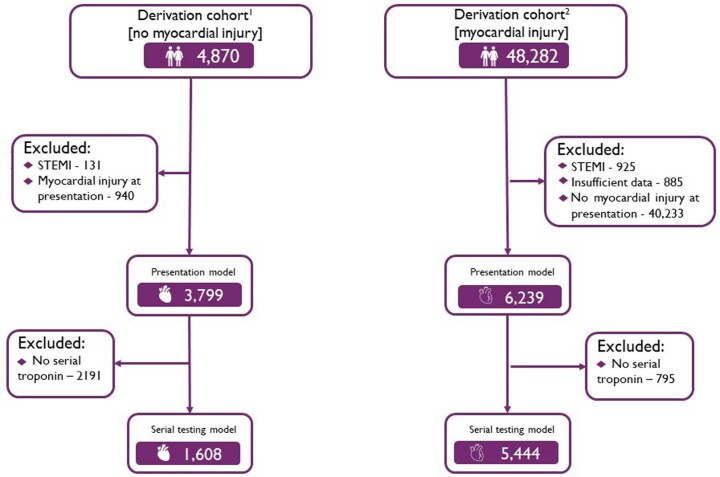
Flow diagram illustrating the populations used to train CoDE-ACS models in patients with and without myocardial injury. ^1^Lancet. 2015 Dec 19;386(10012):2481-8. ^2^Lancet. 2018 Sep 15;392(10151):919–928.

The derivation cohorts together were composed of 10,038 patients (median age 70 years, 48% women) with possible myocardial infarction presenting to 1 of 10 secondary or tertiary care hospitals in Scotland (Table 1 and Extended Data Fig. 1). The ground truth was determined according to the Fourth Universal Definition of Myocardial Infarction^11^ following review of all clinical information and investigations by two clinicians independently, with a third reviewer providing consensus if there was disagreement. The diagnostic outcome was prespecified and included all patients with an adjudicated diagnosis of type 1, 4b or 4c myocardial infarction without ST-segment elevation during the index hospital admission. Models to estimate the probability of myocardial infarction were trained separately in consecutive patients with and without myocardial injury at presentation, defined as a cardiac troponin I concentration above or below the sex-specific 99th percentile upper reference limit on the first measurement. In 6,239 and 3,799 patients with and without myocardial injury at presentation, the final adjudicated diagnosis after serial cardiac troponin measurements was type 1, 4b or 4c myocardial infarction in 3,094 and 132 patients, respectively.

**Table 1:** Baseline characteristics of the derivation and external validation cohorts

	Derivation cohort		External validation cohort	
	No myocardial injury at presentation	Myocardial injury at presentation	No myocardial injury at presentation	Myocardial injury at presentation
Number of patients	3,799	6,239	8,664	1,622
Age, years	62 (50–74)	74 (62–83)	57 (47–69)	70 (59–79)
Sex				
Female	1,580 (42%)	3,199 (51%)	3,048 (35%)	581 (36%)
Male	2,219 (58%)	3,040 (49%)	5,616 (65%)	1,041 (64%)
Chest pain at presentation	3,251 (86%)	4,030 (70%)	8,551 (99%)	1,607 (99%)
Early presenter (≤3 h from symptom onset)	1071 (28%)	1,970 (32%)	3,979 (47%)	549 (34%)
Previous medical conditions				
Myocardial infarction	606 (18%)	837 (13%)	1,804 (21%)	504 (31%)
Ischemic heart disease	1,133 (34%)	2,136 (34%)	2,447 (28%)	657 (41%)
Cerebrovascular disease	236 (7%)	626 (10%)	442 (5%)	140 (9%)
Diabetes mellitus	513 (16%)	919 (15%)	1,267 (15%)	376 (23%)
Previous revascularization				
PCI	360 (11%)	560 (9%)	1,792 (21%)	438 (27%)
CABG	178 (5%)	161 (3%)	555 (6%)	187 (12%)
Medications at presentation				
Aspirin	720 (30%)	2,267 (36%)	2,972 (34%)	744 (46%)
Dual antiplatelet therapy^a^	115 (5%)	309 (5%)	1,804 (27%)	506 (42%)
ACE or ARB	745 (31%)	2,681 (43%)	2,474 (36%)	626 (50%)
Beta-blocker	584 (25%)	2,156 (35%)	2,076 (30%)	520 (41%)
Electrocardiogram result^b^				
Normal	3,279 (87%)	3,308 (64%)	2,947 (44%)	759 (63%)
Myocardial ischemia	458 (14%)	1,351 (26%)	797 (9%)	599 (37%)
ST-segment elevation	93 (3%)	196 (4%)	88 (1%)	20 (2%)
Physiological parameters				
Heart rate, beats per min	77 (65–90)	81 (68–99)	74 (65–85)	77 (66–90)
Systolic blood pressure, mm Hg	137 (121–153)	138 (120–157)	140 (125–156)	142 (127–160)
Hematology and clinical chemistry measurements				
Hemoglobin, g l^−1^	NA	133 (118–146)	143 (133–153)	140 (126–152)
eGFR, ml min^−1^ 1.73 m^2^	86 (69–99)	66 (44–85)	87 (72–96)	73 (55–89)
Presentation high-sensitivity cardiac troponin I, ng l^−1^	3 (2–7)	85 (41–320)	3 (2–6)	144 (53–614)
Serial high-sensitivity cardiac troponin I, ng l^−1^	4 (2–8)	170 (51–1,422)	3 (2–6)	200 (61–894)
Peak high-sensitivity cardiac troponin I, ng l^−1^	5 (3–11)	209 (53–1,786)	3 (2–7)	263 (61–1,118)
Adjudicated diagnosis				
Type 1, 4b or 4c myocardial infarction	132 (3%)	3,094 (49%)	267 (3%)	1,032 (64%)
Type 2 myocardial infarction	33 (1%)	802 (13%)	132 (2%)	169 (10%)
Nonischemic myocardial injury	21 (1%)	2,343 (38%)	180 (2%)	252 (16%)

Values are median (interquartile range) or *n* (percentage). ACE, angiotensin converting enzyme; ARB, angiotensin receptor blockers; CABG, coronary artery bypass grafting; eGFR, estimated glomerular filtration rate; NA, not applicable; PCI, percutaneous coronary intervention.

^a^Two medications from aspirin, clopidogrel, prasugrel or ticagrelor.

^b^Includes warfarin or novel oral anticoagulants.

### Diagnostic performance of cardiac troponin thresholds

**Fig. 1:**
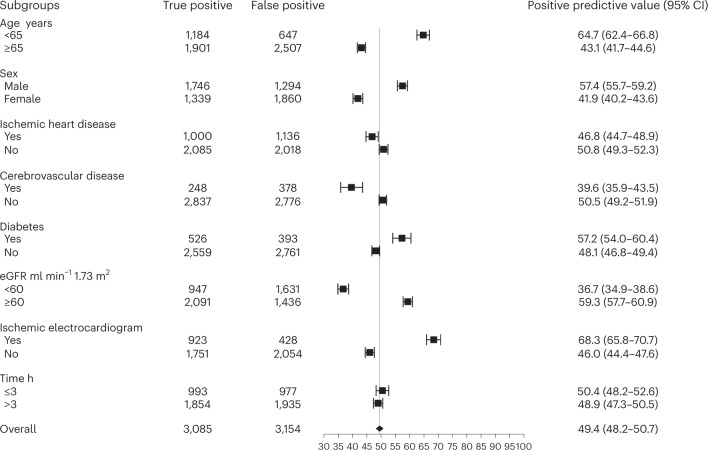
Positive predictive value of the sex-specific 99th percentile cardiac troponin threshold in the derivation cohort across patient subgroups. Data are presented as a central estimate with 95% CIs based on the Clopper–Pearson method. eGFR, estimated glomerular filtration rate.

**Extended Data Fig. 2:**
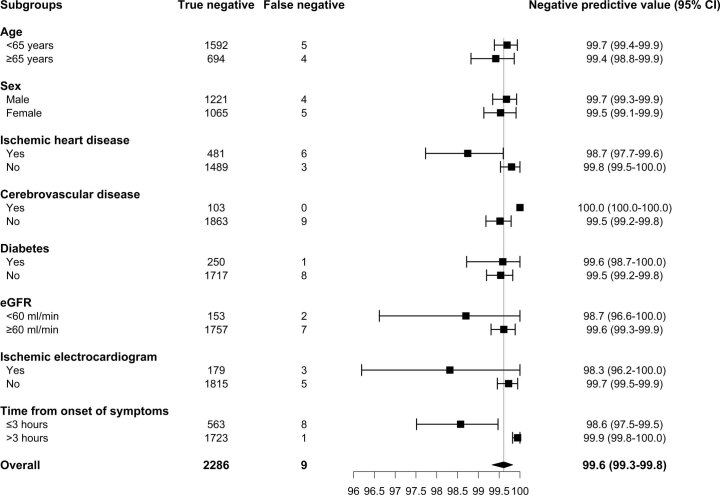
Negative predictive value of the 5 ng/L risk stratification threshold at presentation in the derivation cohort across patient subgroups. Data are presented as a central estimate with 95% confidence intervals based on the Clopper-Pearson method.

In patients without myocardial injury, the negative predictive value of the rule-out threshold of less than 5 ng l^−1^ at presentation was 99.6 (95% confidence interval (95% CI), 99.3–99.8) (Supplementary Table 1). The negative predictive value was lower in patients presenting within 3 h of symptom onset (Extended Data Fig. 2). Among patients with myocardial injury at presentation, the positive predictive value of the sex-specific 99th percentile upper reference limit was 49.4 (95% CI, 48.2–50.7). There was significant heterogeneity in all subgroups, with a lower positive predictive value in those older than 65 years old, in women and in those with ischemic heart disease and impaired renal function (Fig. 1).

### Training and internal validation of models

**Extended Data Fig. 3:**
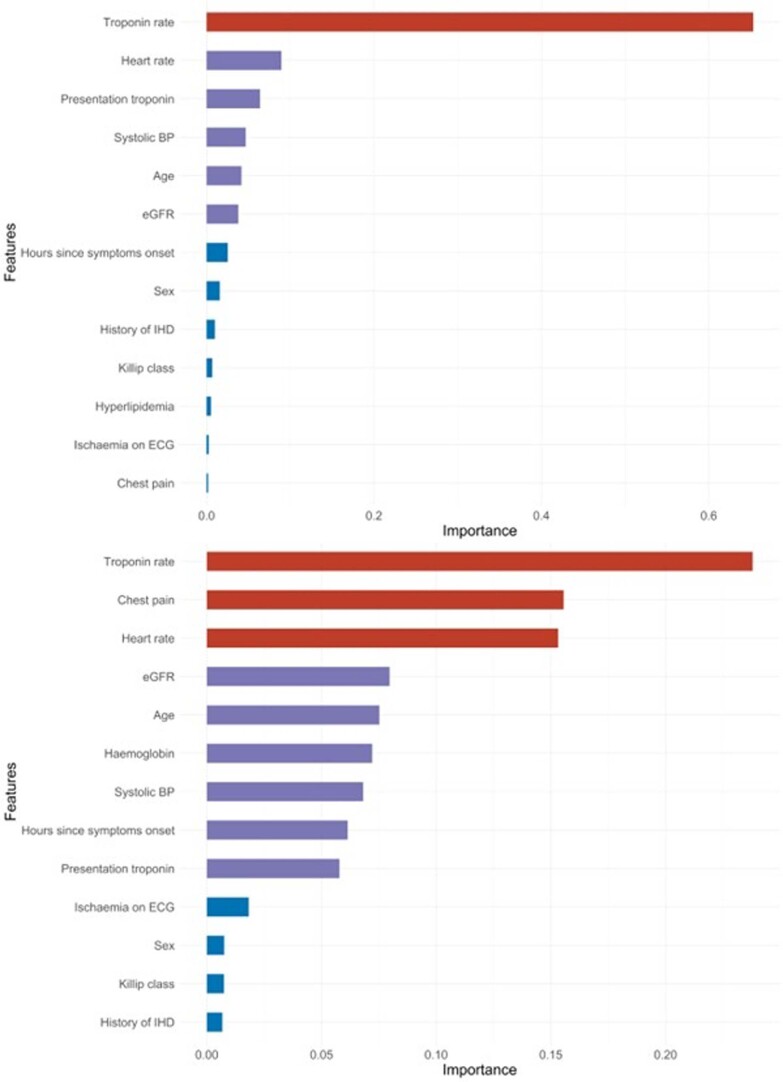
Importance permutation rank of the features in the XGBoost model. (**a**) In patients without myocardial injury. (**b**) In patients with myocardial injury.

An XGBoost model was the best-performing model in patients with and without myocardial injury at presentation and when using the first cardiac troponin measurement or serial measurements (Supplementary Table 2). These XGBoost models were combined within a single clinical decision support system called CoDE-ACS, which computes a score (0–100) corresponding to an individual patient’s probability of myocardial infarction (https://decision-support.shinyapps.io/code-acs/). CoDE-ACS models combine cardiac troponin as a continuous measure with age, sex, time from symptom onset, the presence of chest pain, known ischemic heart disease, hyperlipidemia, heart rate, systolic blood pressure, Killip class, myocardial ischemia on the electrocardiogram, renal function and hemoglobin (Extended Data Fig. 3).

**Extended Data Fig. 4:**
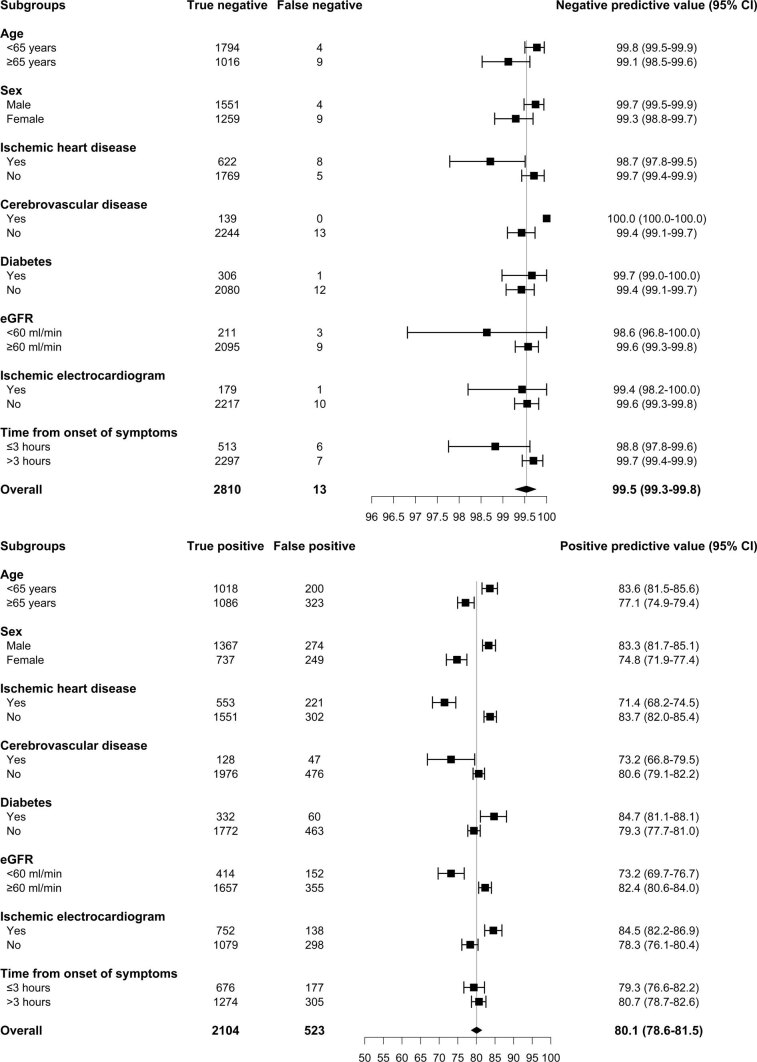
Diagnostic performance of CoDE- ACS scores at presentation in the derivation cohort across patient subgroups. Data are presented as a central estimate with 95% confidence intervals based on the Clopper-Pearson method. (**a**) CoDE-ACS low probability score of less than 3. (**b**) CoDE-ACS high probability score of 61 or more.

In patients without myocardial injury at presentation, a CoDE-ACS score of less than three met our prespecified diagnostic performance criteria with a negative predictive value of 99.5 (99.3–99.8) and sensitivity of 90.2 (84.7–95.0). In those with myocardial injury, a CoDE-ACS score of 61 or more met our prespecified diagnostic performance criteria with a positive predictive value of 80.1 (78.5–81.6) and specificity of 83.4 (82.1–84.7). These scores identifying patients at low and high probability of myocardial infarction performed consistently across subgroups (Extended Data Fig. 4).

**Extended Data Fig. 5:**
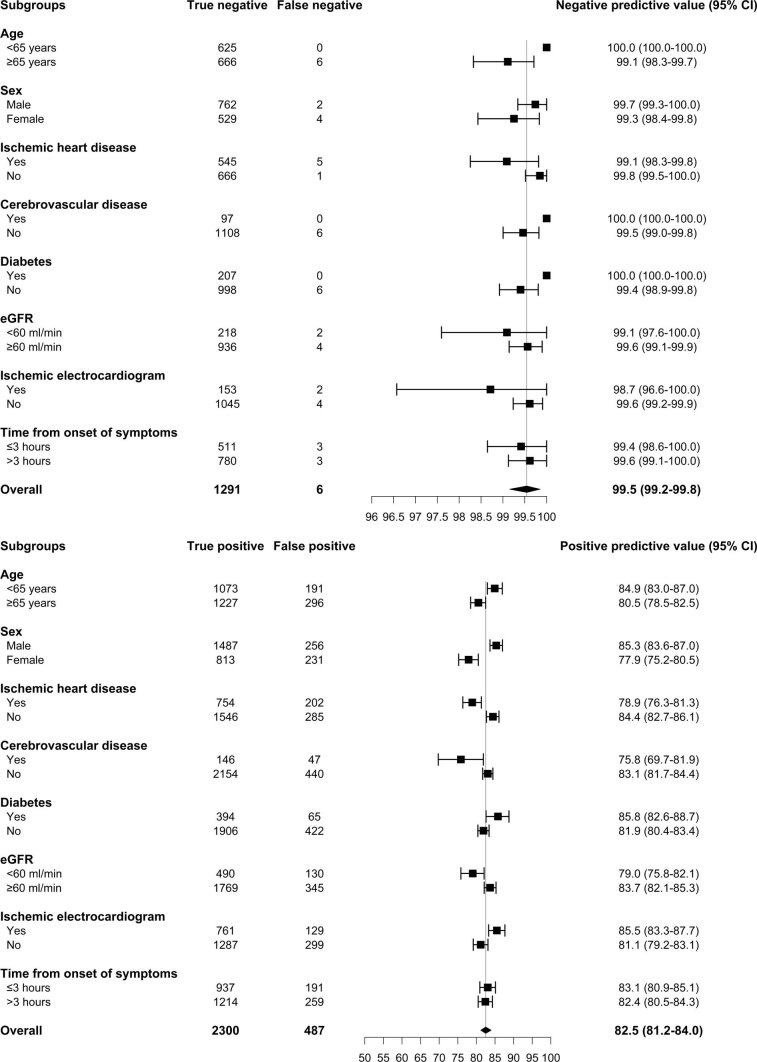
Diagnostic performance of CoDE- ACS scores on serial troponin testing in the derivation cohort across patient subgroups. Data are presented as a central estimate with 95% confidence intervals based on the Clopper-Pearson method. (**a**) CoDE-ACS low probability score of less than 3. (**b**) CoDE-ACS high probability score of 61 or more.

When the presentation and first serial measure of cardiac troponin were incorporated within the models, the same scores of less than 3 and 61 or more that identified patients at low and high probability of myocardial infarction at presentation gave a negative predictive value of 99.5 (99.2–99.8) and sensitivity of 95.5 (92.0–98.5) in those without myocardial injury at presentation and a positive predictive value of 82.5 (81.1–83.9) and specificity of 80.1 (78.4–81.6) in those with myocardial injury (Extended Data Fig. 5). The diagnostic performance of these scores in the models incorporating serial measurements was also consistent across patient subgroups.

### External validation

**Fig. 2:**
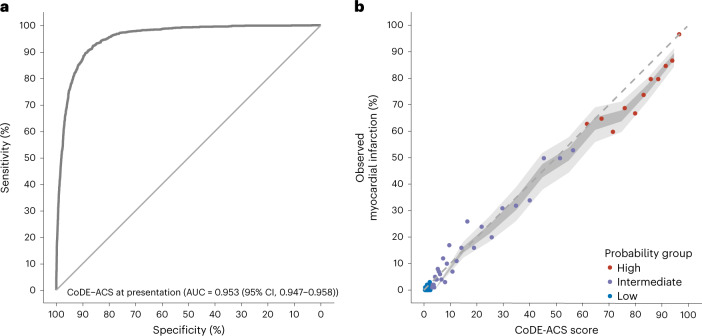
Diagnostic performance of the CoDE-ACS score in the external validation cohort using the presentation troponin concentration alone. **a**, Receiver-operating characteristic curve illustrating the discrimination of the CoDE-ACS for myocardial infarction. **b**, Calibration of the CoDE-ACS score with the observed proportion of patients with myocardial infarction. The dashed line represents perfect calibration. Each point represents 100 patients. Patients are grouped as low (<3), intermediate (3–60) or high probability (≥61) of myocardial infarction. The darker shaded area represents the 95% CI, while the lighter shaded area represents the 99% CI. AUC, area under curve.

**Extended Data Fig. 6:**
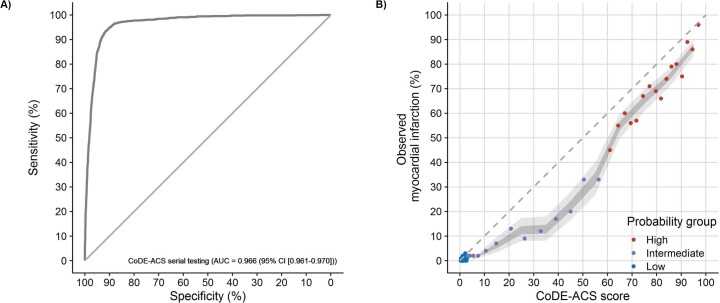
Diagnostic performance of CoDE- ACS in the external validation cohort using serial troponin results. (**a**) Receiver-operating- characteristic (ROC) curve illustrating discrimination of the CoDE-ACS for myocardial infarction. **(b**) Calibration of the CoDE-ACS score with the observed proportion of patients with myocardial infarction. The dashed line represents perfect calibration. Each point represents 100 patients. Patients are grouped as low- (<3), intermediate- (3 to 60) or high-probability (≥61) of myocardial infarction. The darker shaded area represents the 95% confidence interval, while the lighter shaded area the 99% confidence interval.

The external validation cohort consisted of 10,286 patients (median age 60 years, 35% women) with possible myocardial infarction pooled from seven prospective cohort studies enrolling patients across six countries (Table 1). In 8,664 and 1,622 patients with and without myocardial injury at presentation, the final adjudicated diagnosis after serial cardiac troponin measurements was myocardial infarction in 1,032 and 267 patients, respectively. Discrimination of the CoDE-ACS models was excellent, with an area under curve of 0.953 (95% CI, 0.947–0.958) at presentation and 0.966 (95% CI, 0.961–0.970) on serial testing. Similarly, calibration was good using presentation cardiac troponin alone and serial measurements (Brier scores of 0.053 and 0.051, respectively) (Fig. 2 and Extended Data Fig. 6).

### CoDE-ACS pathway compared with cardiac troponin thresholds

In 10,286 patients from the external validation cohort, there was a total of 1,299 (13%) with a final diagnosis of myocardial infarction. When a threshold of less than 5 ng l^−1^ at presentation was applied to those without myocardial ischemia on the electrocardiogram in whom symptom onset was more than 3 h from testing, the proportion ruled out was 27% (2,819 of 10,286). The negative predictive value and sensitivity were 99.7 (95% CI, 99.5–99.8) and 98.3 (95% CI, 97.8–98.6), respectively. When the sex-specific 99th percentile diagnostic thresholds were applied at presentation, the proportion ruled in was 16% (1,622 of 10,286), with a positive predictive value and specificity of 63.6 (95% CI, 62.7–64.5) and 93.4 (95% CI, 92.9–93.9), respectively. The remaining 57% (5,845 of 10,286) of patients had intermediate cardiac troponin concentrations or required serial testing as they presented early or had an abnormal electrocardiogram.

**Fig. 3:**
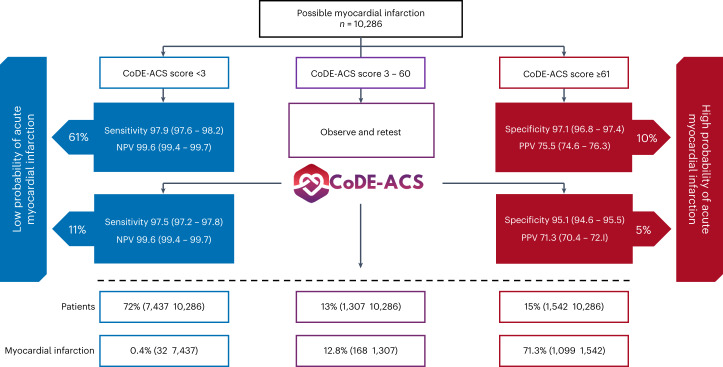
External validation of the performance of the CoDE-ACS pathway in 10,286 patients with possible myocardial infarction. Diagnostic performance of CoDE-ACS models in 10,286 patients from seven international cohorts. Sensitivity, negative predictive value (NPV), specificity and positive predictive value (PPV) with 95% CIs of the CoDE-ACS scores were used to identify patients as low probability (<3) or high probability (≥61) of myocardial infarction at presentation and after serial troponin testing if required.

**Fig. 4:**
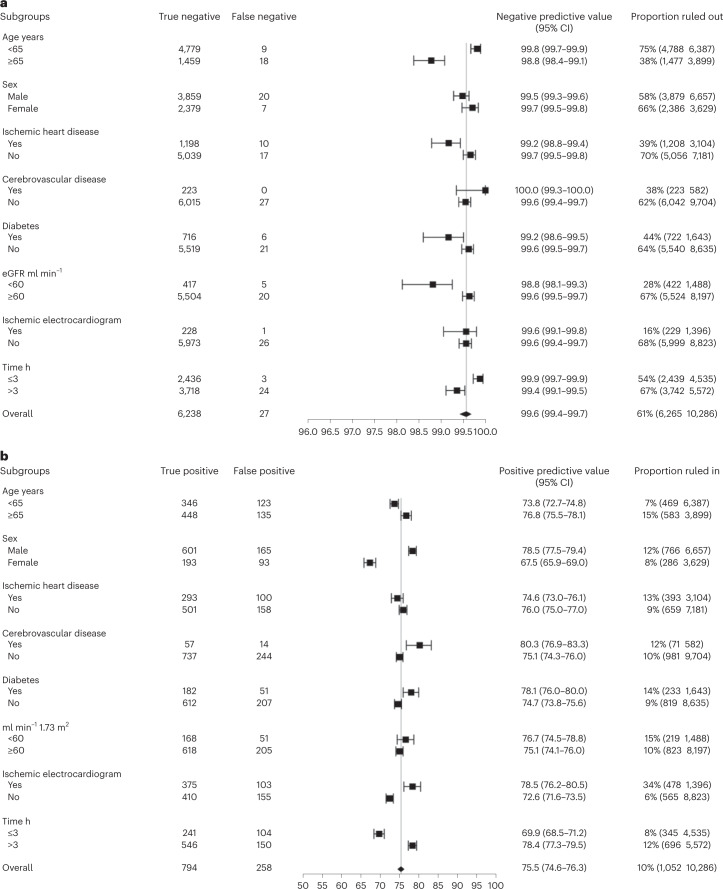
Diagnostic performance of the CoDE-ACS score in the external validation cohort for identifying patients as having a low or high probability of myocardial infarction across patient subgroups. Data are presented as a central estimate with 95% CIs based on the Clopper–Pearson method. **a**, Negative predictive value of the low-probability CoDE-ACS score using the presentation troponin concentration alone across patient subgroups. **b**, Positive predictive value of the high-probability CoDE-ACS score using the presentation troponin concentration alone across patient subgroups. eGFR, estimated glomerular filtration rate.

**Extended Data Fig. 7:**
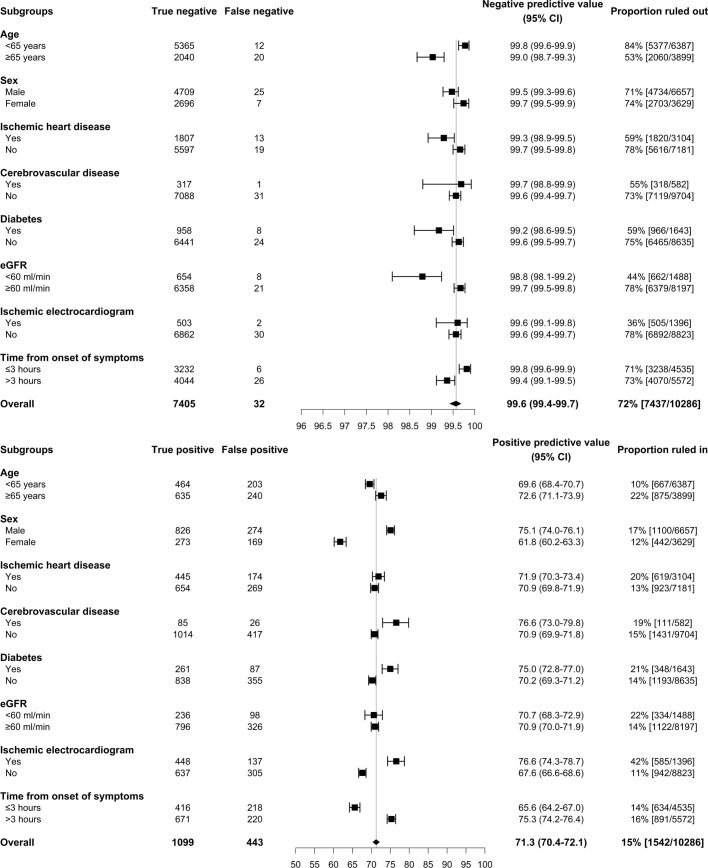
Diagnostic performance of CoDE- ACS scores on serial troponin testing in the external validation cohort across patient subgroups. Data are presented as a central estimate with 95% confidence intervals based on the Clopper-Pearson method. (**a**) CoDE-ACS low probability score of less than 3. (**b**) CoDE-ACS high probability score of 61 or more.

A CoDE-ACS score of less than three identified 61% (6,265 of 10,286) of patients in the external validation cohort as low probability at presentation, with a negative predictive value of 99.6 (95% CI, 99.4–99.7) and a sensitivity of 97.9 (95% CI, 97.6–98.2) (Fig. 3 and Supplementary Tables 3 and 4). A CoDE-ACS score of 61 or greater identified 10% (1,052 of 10,286) of patients at presentation as high probability, with a positive predictive value of 75.5 (95% CI, 74.6–76.3) and a specificity of 97.1 (95% CI, 96.8–97.4) (Fig. 3). Both the low- and high-probability scores performed well in the validation cohort across all subgroups, although there was some heterogeneity observed by age and sex (Fig. 4 and Extended Data Fig. 7).

There were 2,969 (29%) patients in the validation cohort with a CoDE-ACS score of 3–60 at presentation in whom serial testing would be recommended. When the first serial measure of cardiac troponin at any time point was incorporated, a CoDE-ACS score of less than 3 and 61 or greater identified a further 1,172 (11%) and 490 (5%) patients as low and high probability, respectively. Overall, this resulted in 72% (7,437 of 10,286) of patients being identified as low probability with a negative predictive value of 99.6 (95% CI, 99.4–99.7) and sensitivity of 97.5 (95% CI, 97.2–97.8) and 15% (1,542 of 10,286) of patients being identified as high probability with a positive predictive of 71.3 (95% CI, 70.4–72.1) and specificity of 95.1 (95% CI, 94.6–95.5), respectively (Fig. 3 and Supplementary Table 3). After two cardiac troponin tests, the probability remained intermediate in 1,342 (13%) patients, but individual CoDE-ACS scores along with diagnostic metrics associated with those scores are provided within the clinical decision support system and could be used to select patients for further inpatient assessment or outpatient follow-up.

**Extended Data Fig. 8:**
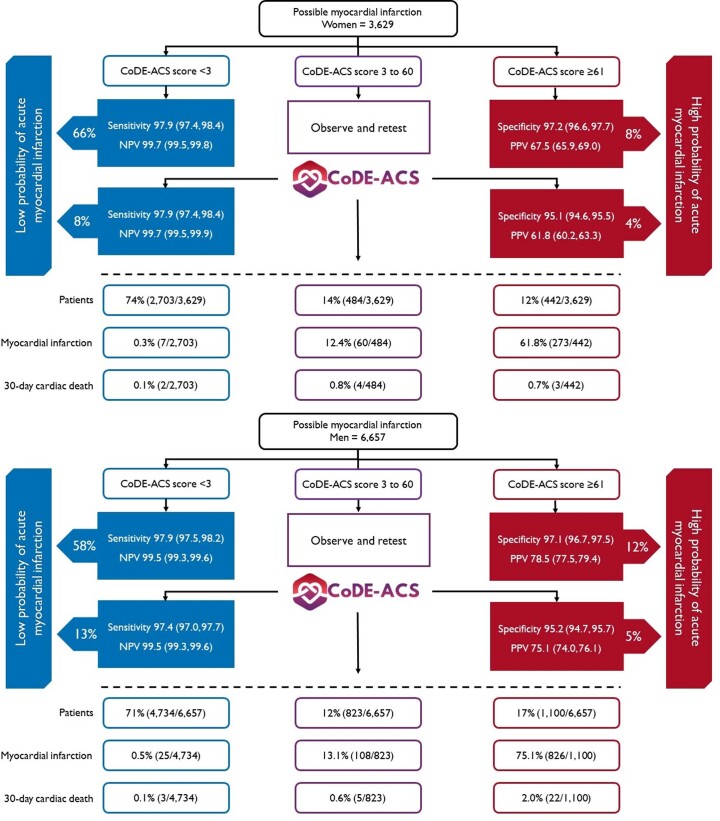
External validation of the performance of the CoDE-ACS pathway in 3,629 women (**a**) and 6,657 men (**b**) with possible myocardial infarction.

**Extended Data Fig. 9:**
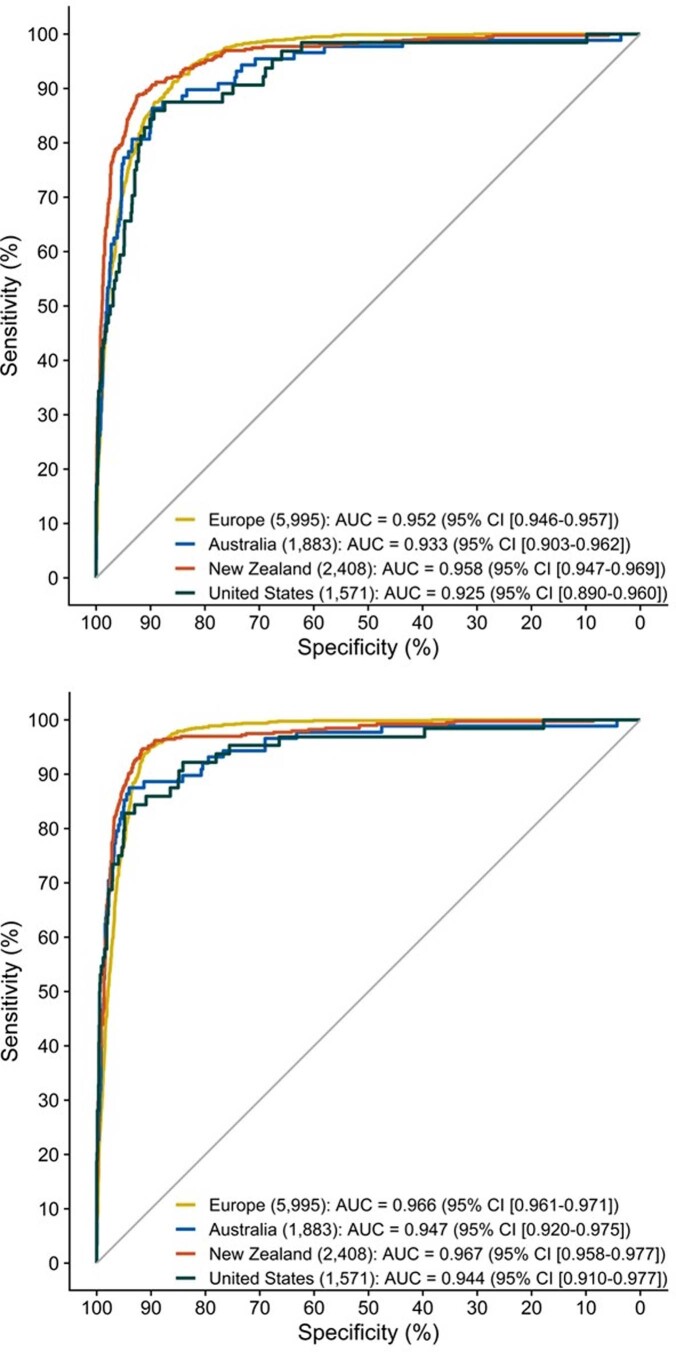
Diagnostic performance of the CoDE-ACS score in the external validation cohorts by region (Europe, Australia, New Zealand and United States). Receiver-operating-characteristic (ROC) curve illustrating discrimination of the CoDE-ACS for myocardial infarction. (**a**) Using the presentation cardiac troponin measurement. (**b**) Using the serial cardiac troponin measurement.

In a series of post hoc analyses, the CoDE-ACS pathway was also validated in a cohort from the US, where the prevalence of myocardial infarction is lower, compared with serial cardiac troponin measurements with relative change criteria and evaluated separately in women and men. In a US cohort of 1,571 patients in whom 64 (4%) had a diagnosis of myocardial infarction, the pathway identified 49% (73 of 1,571) of patients as low probability at presentation with a similar negative predictive value of 99.9 (95% CI, 99.5–100) and a sensitivity of 98.4 (95% CI, 97.7–98.9) and 2% (39 of 1,571) of patients as high probability with a lower positive predictive value of 61.5 (95% CI, 59.1–63.9) but a similar specificity of 99.0 (95% CI, 98.4–99.4) compared with the external validation cohort (Supplementary Table 5). CoDE-ACS was compared with serial cardiac troponin measurements using a relative increase of 20% where the initial value is above the 99th percentile and a relative increase of 50% when it is below the 99th percentile. In the external validation cohort, a CoDE-ACS score of 61 or greater identified more patients as high probability compared with these criteria (15% (1,542 of 10,286) versus 10% (995 of 10,286)) with a higher positive predictive value (71.3 (95% CI, 70.4–72.1) versus 67.4 (95% CI, 66.5–68.3)) and similar specificity (Supplementary Table 6). The CoDE-ACS pathway was evaluated in 3,629 women and 6,657 men from the external validation cohort separately (Extended Data Fig. 8). Performance of the low-probability score and effectiveness were similar in women and men, but the positive predictive value of the high-probability score was lower in women at 67.5 (95% CI, 65.9–69.0) compared with 78.5 (95% CI, 77.5–79.4) in men. Despite differences in disease prevalence between the studies used for external validation, CoDE-ACS performed well across different health care settings (Extended Data Fig. 9). A sensitivity analysis was performed in the external validation cohort, reporting the performance of the CoDE-ACS pathway for a broader diagnostic outcome of type 1, 4b, or 4c or type 2 myocardial infarction (Supplementary Table 7).

### CoDE-ACS pathway compared with other pathways

**Extended Data Fig. 10:**
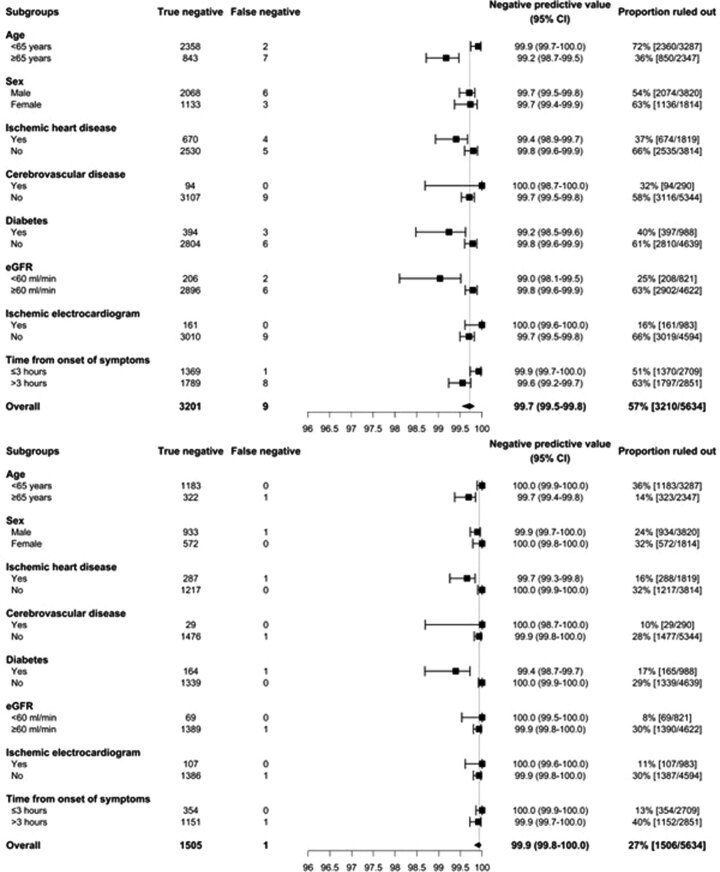
Diagnostic performance in 5,634 patients of the external validation cohort who had cardiac troponin measurements at presentation and 1 hour to enable (A) CoDE-ACS score to identify patients as low-probability of myocardial infarction and (B) the 0/1-hour pathway to rule out myocardial infarction at presentation in subgroups. Data are presented as a central estimate with 95% confidence intervals based on the Clopper-Pearson method.

In our external validation cohort, 5,634 patients had cardiac troponin measurements at presentation and 1 h to enable a comparison of the CoDE-ACS and 0/1-h pathways, with 774 (14%) having a final diagnosis of myocardial infarction (Supplementary Table 8). CoDE-ACS identified twice as many patients as low probability as the 0/1-h pathway at presentation (57 versus 27%) for a similar negative predictive value (99.7 (95% CI, 99.5–99.8) versus 99.9 (95% CI, 99.8–100)) (Extended Data Fig. 10). CoDE-ACS identified a similar proportion of patients as high probability as the 0/1-h pathway at presentation (12 versus 13%) for a higher positive predictive value (67.8 (95% CI, 66.6–69.0) versus 62.3 (95% CI, 61.0–63.5)). When serial measures at 0 and 1 h were incorporated, the CoDE-ACS pathway identified fewer patients as intermediate probability than the 0/1-h pathway (14 versus 29%).

In our external validation cohort, 2,271 patients had the required clinical features and cardiac troponin measurements at presentation and 3 h to enable a comparison of the CoDE-ACS and History, Electrocardiogram (ECG), Age, Risk Factors, and Troponin (HEART) pathways, with 360 (16%) having a final diagnosis of myocardial infarction (Supplementary Table 9). The HEART pathway does not rule out any patients at presentation, whereas CoDE-ACS identified 51% (1,169 of 2,271) as low probability with a negative predictive value of 99.6 (95% CI, 99.2–99.8). At 3 h, CoDE-ACS identified four times as many patients at low probability as the HEART pathway (66 versus 16%) for a similar negative predictive value (99.7 (95% CI, 99.3–99.8) versus 100 (95% CI, 99.8–100)). The positive predictive value of the high-risk criteria in the HEART pathway was significantly lower than the high-probability score from the CoDE-ACS pathway (19.0 (95% CI, 17.4–20.6) versus 69.7 (95% CI, 67.7–71.5)).

Pathways that incorporate machine learning models are more flexible than those using fixed cardiac troponin thresholds or risk scores, allowing health care systems to apply different criteria to define low and high probability of myocardial infarction. For example, a pathway incorporating a lower CoDE-ACS score of two will identify fewer patients as low probability of myocardial infarction at presentation than one using a score of three (50 versus 61%) for a higher negative predictive value (99.7 (95% CI, 99.6–99.8) versus 99.6 (95% CI, 99.4–99.7)) and sensitivity (98.8 (95% CI, 98.6–99.0) versus 97.9 (95% CI, 97.6–98.2)) (Supplementary Table 10).

### Outcomes stratified by the CoDE-ACS score

**Fig. 5:**
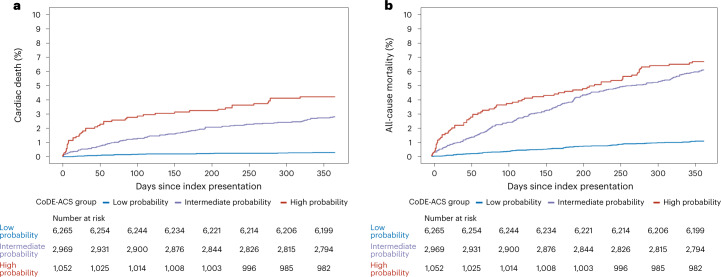
Cumulative incidence of cardiac death and all-cause mortality as stratified by the CoDE-ACS score at presentation in the external validation cohort. **a**,**b**, Data for cardiac death (**a**) and all-cause mortality (**b**).

At 1 year, there were 144 (1.4%) deaths from a cardiac cause and 317 (3.1%) deaths of any cause in the external validation cohort. Compared with patients identified by CoDE-ACS at presentation as intermediate or high probability, those who were low probability of myocardial infarction had a lower rate of cardiac death and all-cause death at 30 days (cardiac death: 0.1 versus 0.5 and 1.8%; all-cause death: 0.1 versus 0.9 and 2.0%, respectively) and at 1 year (cardiac death: 0.3 versus 2.8 and 4.2%; all-cause death: 1.1 versus 6.1 and 6.7%, respectively; log-rank test *P* < 0.001) (Fig. 5).

## Discussion

In patients presenting with possible acute myocardial infarction, we developed and validated the CoDE-ACS clinical decision support system using machine learning with single or serial high-sensitivity cardiac troponin measurements to inform the probability of acute myocardial infarction.

Compared with guideline-recommended pathways using cardiac troponin thresholds and risk scores, CoDE-ACS identified twice as many patients as low probability of myocardial infarction at presentation with a similar negative predictive value and fewer patients as high probability with an improved positive predictive value. Unlike recommended cardiac troponin thresholds, CoDE-ACS scores performed well in subgroups, including men and women, older persons, those with renal impairment or those who present early following the onset of symptoms. We validated performance across multiple health care systems, where the prevalence of myocardial infarction varied from 4 to 16%, and propose a pathway that identifies up to two thirds of patients as low probability of myocardial infarction with a single troponin test and improves the recognition of those with elevated cardiac troponin concentrations who have acute myocardial infarction. While our models were trained to estimate the probability of myocardial infarction during the index hospital admission, patients who were identified as low probability of myocardial infarction were also at low risk of death following discharge, with fewer than 1 in 300 having a cardiac death at 1 year. If adopted in practice, the CoDE-ACS clinical decision support system could reduce time spent in emergency departments, prevent unnecessary hospital admission in patients unlikely to have myocardial infarction and at low risk of cardiac death, and improve the recognition and treatment of those with myocardial infarction rather than myocardial injury, with benefits for both patients and health care providers.

Our study has directly benefited from a substantial body of prior research describing the relationship between cardiac troponin and coronary heart disease, which has transformed the assessment of patients with possible myocardial infarction^26–33^. In particular, approaches harnessing high-sensitivity assays that can quantify cardiac troponin at concentrations well below the diagnostic threshold for myocardial infarction and pathways incorporating risk scores^34^ have been instrumental in improving care^1–5,10^. The use of statistical modeling to guide clinical decisions represents a logical progression of this field and has several important advantages over prior approaches using fixed troponin thresholds or risk scores alone. First, cardiac troponin is known to be influenced by age, sex and renal function^21–24^. Our findings from an unselected cohort of consecutive patients demonstrate marked heterogeneity in the performance of the diagnostic threshold across these groups that was minimized when a model incorporating these features was applied. Second, patients with different symptoms, comorbidities and risk factors have a different pretest probability of having nonischemic myocardial injury or myocardial infarction^35–37^. Incorporating these features into the CoDE-ACS models rather than considering them in isolation as applied in the HEART pathway significantly improved the positive predictive value of an elevated cardiac troponin for myocardial infarction compared with using the same fixed troponin threshold in all patients irrespective of pretest probability. Third, current national and international guidelines recommend serial cardiac troponin measurements in all patients who present within 3 h of symptom onset^11–13^, as it takes time following an episode of myocardial ischemia for cardiac troponin to increase above the thresholds recommended to rule out myocardial infarction^38,39^. CoDE-ACS, by incorporating time from symptom onset, enables early presenters to be ruled out using a single test. Finally, current pathways recommend fixed time points for serial measurements in those who have intermediate cardiac troponin concentrations, which can be challenging to implement in routine practice and may unnecessarily increase the duration of stay. Previous studies have shown that between one in five and one in three patients do not undergo cardiac troponin testing in accordance with pathway recommendations^10,40^. In the 29% of patients not identified as low or high probability using a single cardiac troponin measurement, CoDE-ACS, by incorporating information on the time of testing, permits a second measurement to be incorporated at a flexible time point. The CoDE-ACS pathway incorporating a serial measurement at a flexible time point reduced the proportion of patients requiring further observation and testing twofold and will reduce the potential for harm due to nonadherence with the timing of the serial measurement that is inherent to current diagnostic pathways.

The CoDE-ACS clinical decision support system was influenced by pioneering early studies^41^ and represents a substantial advance on our previous work^42^. The Troponin-Only Manchester Acute Coronary Syndromes score combines cardiac troponin T concentrations at presentation with other clinical observations using logistic regression to identify a third of patients with suspected acute coronary syndrome as low risk of major adverse cardiac events^41^. The myocardial–ischemic–injury index (MI^3^) uses gradient boosting to compute a probability of myocardial infarction but has several limitations. First, while CoDE-ACS ruled in or ruled out myocardial infarction in 71% of patients with a single cardiac troponin test, MI^3^ requires two measurements in all patients to estimate probability. In practice, this would significantly limit the effectiveness of MI^3^ given that accelerated diagnostic pathways in use today enable decisions based on a single cardiac troponin measurement and have been shown to be safe and to prevent unnecessary admissions^8,10,40^. Second, the MI^3^ score was calculated using only age, sex and cardiac troponin concentrations. Although the use of a limited number of variables is laudable for its simplicity, by not including other important features that influence cardiac troponin, the positive predictive value and specificity were lower in patients with comorbidities^43^. Finally, MI^3^ was developed in a small cohort of selected patients, and when we performed a validation in unselected consecutive patients, we observed that calibration was poor, particularly for those at intermediate probability^43^. CoDE-ACS overcomes these limitations by estimating probability using a single cardiac troponin measurement, including other features that influence cardiac troponin concentrations and pretest probability, and by training the model in a large unselected patient population.

The advantage of using machine learning models within a clinical decision support system over fixed cardiac troponin thresholds to generate a probability of myocardial infarction and the diagnostic metrics associated with this probability is that health care systems can apply a decision support system more flexibly. For example, in a health care setting that is more conservative, a lower CoDE-ACS score to identify patients as being at very low probability of myocardial infarction with a negative predictive value of 99.8 and false-negative rate of 1 in 500 could be applied to guide discharge in 50% of patients with a single test. Alternatively, in health care settings where capacity in the emergency department is limited, a lower CoDE-ACS score to identify those as high probability could be applied to reduce the proportion of patients considered of intermediate probability who require observation and serial testing within the department. Our clinical decision support system provides users with the option to select the diagnostic parameters and therefore, the CoDE-ACS score to define low and high probability in order to create a pathway that is optimal for patient flow according to local clinical priorities (https://decision-support.shinyapps.io/code-acs/). In the future, it may be possible to integrate CoDE-ACS with other machine learning approaches using the 12-lead electrocardiogram to further refine performance and reduce the proportion of patients requiring observation^44^. Likewise, the inclusion of findings from other investigations could help our models learn to differentiate between type 1 and type 2 myocardial infarction.

While CoDE-ACS may enable a more flexible approach to the interpretation of cardiac troponin results and therefore, the correct triage of patients in practice, we continue to advocate the use of a sex-specific 99th percentile as the diagnostic threshold for myocardial infarction. Indeed, this threshold was used to adjudicate all cases of myocardial infarction in our derivation and validation cohorts^25,45–49^. However, we recognize the limitations of applying a fixed threshold derived from a reference range population to individual patients who may not be represented in these cohorts. Despite incorporating sex into the CoDE-ACS models, the positive predictive value of the high-probability score was lower in women than men. This may represent true biological differences in the probability of myocardial infarction in women and men or unintended selection bias when enrolling patients into the external validation cohorts. Ultimately, myocardial infarction is a clinical diagnosis that requires judgment to interpret the presenting symptoms and signs and findings from troponin testing and cardiac imaging. We anticipate that use of machine learning models within the CoDE-ACS clinical decision support system will augment rather than replace this clinical judgment and minimize inequalities in care.

Several limitations merit consideration. First, the CoDE-ACS models have been trained and validated using a high-sensitivity cardiac troponin I assay from a single manufacturer. Given that cardiac troponin assays are not standardized across different manufacturers, CoDE-ACS will need to be retrained and validated for other assays. Second, confirmation bias may in part explain the excellent performance of the CoDE-ACS models as they incorporate features that are integral to the diagnosis of myocardial infarction. This was minimized as the ground truth was defined prior to the development of the CoDE-ACS model and does not make CoDE-ACS any less useful as an objective measure of probability in practice. Third, there were important differences in the characteristics of patients enrolled in our derivation and validation cohorts, which likely reflect differences in study inclusion and exclusion criteria, disease prevalence and health care system factors. Despite these differences, CoDE-ACS performed well in different health care settings. However, the enrollment of consented patients rather than unselected patients may have introduced some selection bias, with overrepresentation of younger male patients responsible for the less consistent performance of CoDE-ACS across some subgroups in the validation cohort. Despite this heterogeneity, the false-negative rate was less than 1 in 100 across subgroups, even in those with increased pretest probability of myocardial infarction. We acknowledge that in high-risk subgroups, such as those older than 65 years old or with prior ischemic heart disease or renal impairment, additional prospective validation would be useful. No decision support system or pathway should be used without consideration of pretest probability and clinical judgment. In our application, the predictive values are reported alongside the score for individual patients, so clinicians can use this information to guide care. Fourth, although our evaluation included participants from across seven countries, the majority were White, and therefore, we were not able to evaluate whether diagnostic performance was consistent across different ethnic groups. Finally, CoDE-ACS was validated in cohorts that had completed enrollment, and care was not guided by our clinical decision support system. Prospective validation and an evaluation of the impact of providing diagnostic probabilities and decision support on management following implementation of CoDE-ACS into practice are warranted.

In conclusion, we have developed a clinical decision support system using machine learning with single or serial high-sensitivity cardiac troponin measurements to inform the probability of acute myocardial infarction. CoDE-ACS was superior to pathways that use fixed cardiac troponin thresholds or risk scores and performed consistently across different health care systems and patient subgroups. We propose a care pathway that identifies more patients as low probability of myocardial infarction with a single cardiac troponin test and improves the recognition of those with myocardial infarction compared with the current standard of care. If adopted in practice, CoDE-ACS could reduce time spent in emergency departments, prevent unnecessary hospital admissions and improve the early treatment of myocardial infarction, with benefits for both patients and health care providers.

## Methods

### Study population

The High-STEACS trial population was used for the derivation of the CoDE-ACS models. As previously described, High-STEACS was a stepped-wedged cluster-randomized, controlled trial to evaluate the implementation of a high-sensitivity cardiac troponin I assay in consecutive patients with suspected acute coronary syndrome presenting to 10 secondary and tertiary hospitals in Scotland between 10 June 2013 and 3 March 2016 (ref. ^25^).

Patients were included in this prespecified secondary analysis (Supplementary Note 1) based on the following criteria: (1) age ≥18 years old, (2) presentation with suspected acute coronary syndrome, (3) cardiac troponin measured using the ARCHITECT_STAT_ high-sensitivity cardiac troponin I assay (Abbott Laboratories) and (4) availability of electrocardiographic and physiological data for diagnostic adjudication. Patients with a diagnosis of ST-segment elevation myocardial infarction were excluded given they undergo coronary revascularization directly without troponin testing in the emergency department (Extended Data Fig. 1).

### Adjudication of the ground truth and outcomes

The model was trained to identify patients with an adjudicated diagnosis of type 1, type 4b or type 4c myocardial infarction during the index hospital admission. The ground truth was adjudicated according to the Fourth Universal Definition of Myocardial Infarction by two clinicians independently, with a third reviewer providing consensus if there was disagreement^11,25,50^. All diagnoses were adjudicated where there was evidence of myocardial injury at presentation or on serial testing defined as any high-sensitivity cardiac troponin I concentration above the sex-specific 99th percentile. Type 1 myocardial infarction was defined as myocardial necrosis (any high-sensitivity cardiac troponin I concentration above the 99th percentile with a rise and/or fall in concentration where serial testing was performed) in the context of a presentation with possible myocardial infarction due to either symptoms or signs of myocardial ischemia on the electrocardiogram. Patients with symptoms or signs of myocardial ischemia due to increased oxygen demand or decreased supply (for example, tachyarrhythmia, hypotension or anemia) secondary to an alternative condition or a coronary mechanism other than atherothrombosis and myocardial necrosis were defined as having type 2 myocardial infarction. Types 4b and 4c myocardial infarction were defined where myocardial ischemia and myocardial necrosis were associated with stent thrombosis or restenosis, respectively, on coronary angiography. Regional and national registries were used to follow patients for 1 year. The cause of death was adjudicated by investigators masked to troponin concentrations during the index presentation.

### Performance of guideline-recommended cardiac troponin thresholds

We evaluated the diagnostic performance and proportion of patients identified by guideline-recommended cardiac troponin thresholds to rule out (5 ng l^−1^) and rule in (99th percentile of 16 ng l^−1^ (women) and 34 ng l^−1^ (men)) myocardial infarction^21^. These were evaluated in the overall population and in prespecified subgroups by age, sex, time from symptom onset to troponin measurement, renal impairment, prior ischemic heart disease, diabetes mellitus, cerebrovascular disease and ischemia on the electrocardiogram.

### Feature selection and processing

We used high-sensitivity cardiac troponin I concentrations as a continuous measure. We selected 12 objective clinical variables known to be associated with cardiac troponin concentration and pretest probability of myocardial infarction or to aid in the discrimination of myocardial injury from infarction that were found to have the highest relative importance in our model training phase. These were age, sex, the number of hours from symptom onset to cardiac troponin measurement, chest pain, known ischemic heart disease, hyperlipidemia, heart rate, systolic blood pressure, Killip class, evidence of myocardial ischemia on the electrocardiogram, renal function (estimated glomerular filtration rate calculated using the Chronic Kidney Disease Epidemiology Collaboration formula)^51^ and hemoglobin. To maximize the clinical utility of our models, we first developed models using the cardiac troponin concentration at presentation alone. We subsequently developed models to include a second cardiac troponin concentration measured at an early and flexible time point.

### Model development, selection and external validation

We first developed and evaluated models using four statistical methods—logistic regression, naïve Bayes, random forest and extreme gradient boosting (XGBoost)^52–54^. XGBoost is a supervised machine learning technique initially proposed by Chen and Guestrin^52^. In brief, gradient boosting employs an ensemble technique to iteratively improve model accuracy for regression and classification problems. This ensemble-based algorithm is achieved by creating sequential models using decision trees as learners, where subsequent models attempt to correct errors of the preceding models^53,54^. In the boosting method, individuals who were misclassified by the previous model are assigned a higher weight to increase their chance of being selected in subsequent models. Each model is subsequently fitted in a stepwise fashion to minimize loss function, such as absolute error or squared error (the amount that predicted values differ from the true values). XGBoost refers to the reengineering of gradient boosting to significantly improve the speed of the algorithm by pushing the limits of computational resources. The output of the XGBoost model is a probability that is computed by performing an inverse logit transformation of the sum of the weights of the terminal nodes of the trained model.

The mathematical formula for the gradient boosting model can be described as

1 $$\hat y_{i} = \mathop {\sum}\limits_{k = 1}^K {f_{\mathrm{k}}} \left( {{\mathbf{x}}_{i}} \right),f_{\mathrm{k}} \in F,$$

where *f* is a function that maps each variable vector **x**_*i*_ (**x**_*i*_ = {**x**_*i*_, **x**_2_, …, **x**_*n*_}, *i* = 1, 2, *N*) to the outcome *y*_*i*_, *K* is the number of Classification and Regression Trees (*k* = 1, 2, *N*) and *F* is the space of function containing all Classification and Regression Trees^55^.

XGBoost optimizes an objective function of the form

2 $${\mathrm{Obj}} = \mathop {\sum}\limits_{i = 1}^N l \left( {y_{i},\hat y_{i}} \right) + \mathop {\sum}\limits_{k = 1}^K {{{\Omega }}(f_{\mathrm{k}})}$$

where the first term is a loss function *l*, which evaluates how well the model fits the data by measuring the difference between the prediction *ŷ*_*i*_ and the outcome *y*_*i*_. The second term, the regularization term, is used by XGBoost to avoid overfitting by penalizing the complexity of the model. Furthermore, to improve and fully leverage the advantages of XGBoost, we tuned the hyperparameters of the algorithm defined below through a grid search strategy using 10-fold crossvalidation (Supplementary Table 11).

Given that the features that inform diagnosis differ for ruling in and ruling out myocardial infarction, we developed separate models for those with and without myocardial injury at presentation. Here, myocardial injury was defined as a cardiac troponin I concentration above the sex-specific 99th percentile upper reference limit (16 ng l^−1^ in women and 34 ng l^−1^ in men) on the first measurement^14,21^. Furthermore, given that practice guidelines recommend diagnostic pathways that use a single measure of cardiac troponin to rule in or rule out myocardial infarction, we also trained these models separately using the first cardiac troponin measurement alone and then, incorporating the second serial measurement at a flexible time point, resulting in four separate models for each method. For all models in the derivation cohort, we multiply imputed 10 datasets to account for missing data^56^ and performed 10 iterations of 10-fold crossvalidation to compute a score (0–100) that corresponded to an individual patient’s probability of having myocardial infarction.

We then identified the scores that would classify the highest proportion of patients as high or low probability at prespecified performance criteria for rule in (80% positive predictive value and 80% specificity) myocardial infarction in those with myocardial injury and to rule out (99.5% negative predictive value and 90% sensitivity) myocardial infarction in those without myocardial injury. These criteria were based on prior analysis and an international survey of acceptable risk by physicians^14,42,57^. It is important to highlight that these performance criteria are for the evaluation of scores separately in patients with and without myocardial injury at presentation. When these scores are applied to all patients with possible myocardial infarction, higher sensitivity and specificity would be anticipated and required in practice.

The model with the best discrimination in those without myocardial injury at presentation that identified the largest proportion of patients as low probability according to our prespecified performance criteria was selected and integrated into our CoDE-ACS clinical decision support system (https://decision-support.shinyapps.io/code-acs/).

We externally validated CoDE-ACS in the Advantageous Predictors of Acute Coronary Syndromes Evaluation (APACE), the Improved Assessment of Chest Pain Trial (IMPACT), the 2-Hour Accelerated Diagnostic Protocol to Assess Patients with Chest Pain Symptoms Using Contemporary Troponins as the Only Biomarker (ADAPT), the Emergency Department Assessment of Chest Pain Score (EDACS), the Signal Peptide in Acute Coronary Events (SPACE) and the Use of Abbott High Sensitivity Troponin I Assay in Acute Coronary Syndromes (UTROPIA) cohorts from Switzerland, Spain, Poland, Czech Republic, Australia, New Zealand and the US^45–49,58^. All analyses were performed in R v.4.1.2.

### Description of the cohort studies pooled for external validation

#### APACE

##### Study design and population

APACE was a prospective international multicenter study with 12 centers in five countries aiming to advance the early diagnosis of myocardial infarction (ClinicalTrials.gov registry number NCT00470587). From the 8,267 adult patients (≥18 years) presenting to the emergency department with symptoms suggestive of myocardial infarction, 5,995 were included in the external validation dataset. Cardiac troponin samples from enrollment and on serial testing at 1, 2 or 3 h depending on availability were used for validation of the CoDE-ACS models. While enrollment was independent of renal function, we excluded patients with terminal kidney failure on chronic dialysis. The study was carried out according to the principles of the Declaration of Helsinki and approved by the local ethics committees. Written informed consent was obtained from all patients. For this analysis, patients with an ST-segment elevation myocardial infarction, patients with missing high-sensitivity cardiac troponin I concentrations at presentation and patients in whom the diagnosis remained unknown even after final adjudication with at least one elevated cardiac troponin concentration, thereby possibly indicating myocardial infarction, were excluded.

##### Adjudication and follow-up

Myocardial infarction was defined and cardiac troponin concentrations were interpreted as recommended in current guidelines^59–61^. In brief, myocardial infarction was diagnosed when there was evidence of myocardial injury with a clinically significant rise and/or fall in a clinical setting consistent with myocardial ischemia. Patients with myocardial infarction were further classified into type 1 (primary coronary events) and type 2 (ischemia due to increased demand or decreased supply: for example, tachyarrhythmia or hypertensive urgency)^12,59^. All other patients were classified as unstable angina, noncardiac chest pain, cardiac but noncoronary disease (for example, tachyarrhythmia or myopericarditis) or symptoms of unknown origin with normal concentrations of cardiac troponin.

The adjudication of final diagnoses was performed centrally in the core laboratory (University Hospital Basel) for all patients using the Abbott ARCHITECT high-sensitivity cardiac troponin I assay (Abbott Laboratories). More specifically, two independent cardiologists not directly involved in patient care reviewed all available medical records (including patient history, physical examination, results of laboratory testing (including cardiac troponin concentrations), radiological tests, electrocardiography, echocardiography, cardiac exercise test, lesion severity and morphology in coronary angiography, and the discharge summary) pertaining to the patient from the time of emergency department presentation to 90-day follow-up. In situations of diagnostic disagreement, cases were reviewed and adjudicated in conjunction with a third cardiologist. Sex-specific 99th percentile upper reference limits of the high-sensitivity cardiac troponin I assay (16 ng l^−1^ in women, 34 ng l^−1^ in men) were used to define myocardial injury. Absolute changes in cardiac troponin were used to determine clinically significant changes^62–66^. Based on studies of the biological variation of cardiac troponin^67,68^ as well as on data from previous chest pain cohort studies^62,69^, a clinically significant absolute change was defined as a rise or fall of at least 10 ng l^−1^ within 6 h or in an assumption of linearity, as an absolute change of 6 ng l^−1^ within 3 h. Patients were contacted 3 and 12 months after discharge by telephone calls or in written form. Information regarding death during follow-up was furthermore obtained from the patient’s hospital notes, the family physician’s records and the national registry on mortality.

#### IMPACT

##### Study design and population

IMPACT was an intervention trial on adult patients in the emergency department with potential acute coronary syndrome (ACTRN12611000206921)^47^. In total, 1,366 patients were recruited prospectively between February 2011 and March 2014, while 1,086 were included in the validation dataset. Cardiac troponin samples from enrollment and at 2 h were used for validation of the CoDE-ACS models. The study was approved by the Royal Brisbane and Women’s Hospital Human Research and Ethics Committee (HREC/10/QRBW/403). Informed written consent was obtained from all participants. Recruitment occurred between 0800 and 1700 and included patients aged ≥18 years with at least 5 min of symptoms suggestive of and planned testing for acute coronary syndrome. Research staff identified eligible patients. Patients were excluded if they had a clear nonacute coronary syndrome cause for their symptoms, they were unwilling or unable to provide informed consent (for example, language barrier), staff considered that recruitment was inappropriate (for example, terminal illness), they were transferred from another hospital, they were pregnant, they were recruited to the study within the previous 30 days or they were unable or unwilling to be contacted after discharge.

Risk stratification occurred per the IMPACT protocol. Initial troponin and electrocardiographic testing was performed on presentation. High-risk patients were treated according to the 2006 National Heart Foundation/Cardiac Society of Australia and New Zealand guidelines. Low- and intermediate-risk patients were assessed using an accelerated investigation strategy, with repeat troponin testing 2 h after the first test. Routine inpatient stress testing was recommended only for intermediate-risk patients. Low-risk patients were discharged after normal 0- and 2-h biomarkers, with correspondence to their general practitioner stating that additional objective testing was not indicated. Cardiac troponin was measured by the Beckman Coulter second-generation AccuTnI assay (Beckman Coulter) to guide clinical practice and in stored material using the Abbott ARCHITECT high-sensitivity cardiac troponin I assay (Abbott Laboratories). This clinical assay is a sensitive troponin assay with a coefficient of variation of 14% at the 99th percentile value of 0.04 μg l^−1^ and a 10% coefficient of variation of 0.06 μg l^−1^. Values of >0.04 μg l^−1^ were considered elevated. Blood samples were taken on presentation and 2 h later for low- and intermediate-risk patients and at 0 and 6 h for high-risk patients. All available troponin results were used for clinical decision-making.

##### Adjudication and follow-up

Telephone follow-up occurred 30 days after presentation by research nurses. All information was verified through medical record databases and cardiac investigation results. Outcomes were adjudicated independently by local cardiologists using predefined standardized reporting definitions, with access to the clinical record, electrocardiogram, cardiac troponin measurements and all subsequent investigations from standard care. A second cardiologist conducted a blind review of all acute coronary syndromes and 10% of nonacute coronary syndrome cases. In cases of disagreement between the two adjudicators, end points were agreed on by consensus. Myocardial infarction was defined according to international guidelines and based on evidence of myocardial necrosis and ischemia. Patients with acute myocardial infarction were further subdivided into acute myocardial infarction type 1 (primary coronary events) and acute myocardial infarction type 2 (ischemia due to increased demand or decreased supply: for example, tachyarrhythmias or hypertensive crisis). Myocardial necrosis was defined as a 20% increase or decrease in cardiac troponin concentration with at least one value above the 99th percentile of the normal reference range. Evidence of myocardial ischemia included the electrocardiogram or cardiac imaging.

#### ADAPT-BSN (Brisbane)

##### Study design and population

The ADAPT-BSN trial was a prospective observational validation study designed to assess a predefined accelerated diagnostic pathway that consisted of the TIMI (thrombolysis in myocardial infarction) score risk assessment, electrocardiogram (ECG), and 0- and 2-h central laboratory contemporary cardiac troponin I as the only biomarker. The original study population was from both Brisbane, Australia and Christchurch, New Zealand^48^. From November 2008 to February 2011, a total of 978 unselected patients presenting to the emergency department of the Royal Brisbane and Women’s Hospital with symptoms of possible acute myocardial infarction were recruited, while 797 patients were included in the validation dataset. Cardiac troponin samples from enrollment and 2 h were used for validation of the CoDE-ACS models. Criteria for enrollment included age ≥18 years of age with at least 5 min of symptoms where the attending physician planned to perform serial cardiac troponin tests. Patients were excluded for any of the following: a clear cause other than acute coronary syndrome for the symptoms (for example, examination findings of pneumonia), inability to provide informed consent, staff considered recruitment to be inappropriate (for example, receiving palliative treatment), transfer from another hospital, pregnancy, previous enrollment or inability to be contacted after discharge. Perceived high risk was not used as an exclusion criterion. Written informed consent was obtained from all patients. Patients were managed according to local hospital protocols, including clinical history, physical examination, 12-lead ECG, continuous ECG monitoring, pulse oximetry, standard blood tests and chest radiography. Clinical blood draws for local cardiac troponin measurement were performed at presentation and then, 6–12 h afterward. Management of patients was at the discretion of the attending physician.

##### Adjudication and follow-up

Final diagnoses were adjudicated by independent cardiologists not directly involved in patient care. Adjudication was based on all available medical records (including patient history, physical examination, all laboratory testing (including cardiac troponin levels), radiological testing, electrocardiography, echocardiography, cardiac exercise test, lesion severity and morphology in coronary angiography, and the discharge summary) pertaining to the patient from the time of emergency department presentation to 30-day follow-up. Myocardial infarction was diagnosed when there was evidence of myocardial necrosis with a clinically significant rise and/or fall in a clinical setting consistent with myocardial ischemia. Patients with acute myocardial infarction were further subdivided into type 1 myocardial infarction (primary coronary events) and type 2 myocardial infarction (ischemia due to increased demand or decreased supply). After discharge, patients were contacted after 6 weeks and 12 months (Brisbane) by telephone calls or in written form. Information regarding death was furthermore obtained from the patients’ hospital notes, the family physician’s records and the national registry on mortality.

#### ADAPT-CH (Christchurch)

##### Study design and population

The ADAPT-CH study was prospectively performed in accordance with the ADAPT-BSN study (see above). From the 1,125 patients recruited between February 2011 and March 2014, 1,000 were included in the validation dataset. Cardiac troponin samples from enrollment and 2 h were used for validation of CoDE-ACS models. It aimed to compare the effectiveness of a rapid diagnostic pathway with a standard care diagnostic pathway for the assessment of patients with possible cardiac chest pain in a usual clinical practice setting. Patients in the emergency department, where the attending physician was investigating for possible acute coronary syndrome, were included.

##### Adjudication and follow-up

Two senior clinicians adjudicated for the presence independently for any major adverse cardiac event. A third senior clinician adjudicated any disagreements with the first two clinicians.

#### ADAPT-RCT (Randomised Controlled Trial)

##### Study design and population

The ADAPT-RCT was a single-center randomized parallel-group trial with blinded outcome assessments conducted in an academic general and tertiary hospital (Australia New Zealand Clinical Trials Registry number 12610000766011). Participants included adults with acute chest pain consistent with acute coronary syndrome for whom the attending physician planned further observation and troponin testing in the Emergency Department at Christchurch Hospital, Christchurch, New Zealand. Patient recruitment occurred from 11 October 2010 to 4 July 2012, with a 30-day follow-up. From the 635 patients recruited, 540 were included in the validation dataset.

##### Adjudication and follow-up

Adjudication and follow-up were as described for ADAPT-CH.

#### EDACS

##### Study design and population

EDACS was a pragmatic randomized, controlled trial (Australia New Zealand Clinical Trials Registry number 12613000745741) of adults with suspected acute myocardial infarction. The primary outcome was the proportion of patients discharged to outpatient care within 6 h of attendance without a subsequent major adverse cardiac event within 30 days. There were 558 patients recruited, 279 in each arm. From 558 patients presenting to a single center (Christchurch, New Zealand), 529 were included in the validation cohort.

##### Adjudication and follow-up

Adjudication and follow-up were as described for ADAPT-CH.

#### SPACE

##### Study design and population

For SPACE, patients presenting to Christchurch Hospital with the primary complaint of chest pain of less than 4 h in duration were offered recruitment into our prospective, observational study (http://www.anzctr.org.au, number 12609000057280). Patients with the primary complaint of acute chest, epigastric, neck, jaw or arm pain suspicious of acute coronary syndrome without obvious noncardiac origin lasting ≥20 min were enrolled in accordance with guideline definitions. More general/atypical symptoms (such as fatigue, nausea, vomiting, sweating and faintness) were not used as inclusion criteria, and those on dialysis or with terminal kidney failure were excluded. From the 346, a total of 339 patients were included in the external validation dataset. Blood samples for measurement of high-sensitivity cardiac troponin I (Abbott Laboratories) were taken at 0, 1, 2 and 12–24 h after presentation. Cardiac troponin concentrations from time 0 and 2 h were used for validation of CoDE-ACS models.

##### Adjudication and follow-up

The adjudicated diagnosis of myocardial infarction was made in accordance with the 2012 European Society of Cardiology (ESC)/ American College of Cardiology Foundation (ACCF), American Heart Association (AHA)/ World Heart Federation (WHF) task force guidelines by two independent cardiologists with access to all clinical data from standard care. The biochemical component of the diagnosis of myocardial infarction was made using a late-generation cardiac troponin I assay with one value in the ≥99th percentile upper reference limit (0.03 μg l^−1^) and a rise or fall of 50% of the reference limit (0.015 μg l^−1^) within 12 h of presentation. At 45 and 365 days following discharge, enrolled patients were contacted by telephone or in writing to complete a follow-up interview/questionnaire. Reported clinical events were identified from the patients themselves (or their primary physician) and confirmed by clinical adjudication, centralized New Zealand Ministry of Health database registry entries on mortality and events, and records of the treating institution.

#### UTROPIA

##### Study design and population

UTROPIA was a prospective cohort study enrolling consecutive, unselected patients who presented from 4 February 2014 through 9 May 2014 to the emergency department, in whom serial cardiac troponin I measurements (0, 3, 6 and 9 h) were ordered on clinical indication at Hennepin County Medical Center (NCT02060760) to rule in or rule out acute myocardial infarction. The study protocol was approved by the institutional review committee. For inclusion, patients needed a baseline cardiac troponin I measurement at presentation, at least one additional cardiac troponin I measurement within 24 h of presentation before discharge and at least one 12-lead ECG performed. Exclusion criteria were younger than 18 years old, ST-segment elevation myocardial infarction, pregnancy, trauma, declined to participate, did not present through the emergency department, or the patient was transferred from an outside hospital. For patients with more than one presentation during the study period, only the first presentation was included.

##### Adjudication

All patients with at least one cardiac troponin I measurements above the 99th percentile were adjudicated according to the Third Universal Definition of Myocardial Infarction consensus recommendations by two clinicians following review of all available medical records, including the 12-lead ECG, echocardiography, angiography, cardiac troponin I results and clinical presentation. Patients in whom there was a discrepancy in the diagnosis were reviewed and adjudicated by a third senior clinician.

### Comparison with other pathways

We compared CoDE-ACS with the HEART pathway and the 0/1-h pathway recommended by the European Society of Cardiology. The HEART (History, Electrocardiogram, Age, Risk factors and Troponin) pathway identifies low- and high-probability patients with a HEAR (History, Electrocardiogram, Age, and Risk factors) score of less than or equal to three and negative cardiac troponin concentrations at 0 and 3 h and a HEAR score greater than or equal to four or positive cardiac troponin concentrations at 0 or 3 h, respectively^34^. We used the sex-specific 99th percentile to define positive or negative cardiac troponin concentrations within the HEART pathway. The 0/1-h pathway identifies patients at low risk with either very low cardiac troponin concentrations at presentation or low concentrations in combination with a small absolute change at 1 h. It identifies patients at high risk with either very high cardiac troponin concentrations at presentation or a relevant absolute change at 1 h (ref. ^12^).

### Ethics statement

The High-STEACS trial was registered (ClinicalTrials.gov registry number NCT01852123) and approved by the Scotland A Research Ethics Committee, by the Public Benefit and Privacy Panel for Health and Social Care, and by each National Health Service Health Board^25^. This analysis was prespecified in the trial protocol and was performed according to a separate statistical analysis plan. As the trial intervention was implemented at the hospital level, consent was not sought from individual patients. All data were collected prospectively from the electronic patient record, deidentified and linked to regional and national registries in a data repository within a Secure Data Environment (DataLoch). All cohort studies contributing to the external validation were approved by their respective local research ethics committee or institutional review board with written informed consent from participants.

### Reporting summary

Further information on research design is available in the Nature Portfolio Reporting Summary linked to this article.

## Online content

Any methods, additional references, Nature Portfolio reporting summaries, source data, extended data, supplementary information, acknowledgements, peer review information; details of author contributions and competing interests; and statements of data and code availability are available at 10.1038/s41591-023-02325-4.

## Supplementary information

Supplementary Information Supplementary Figs. 1–10 and Tables 1–11.

Reporting Summary

Supplementary Note 1 Checklist for the supervised clinical ML study.

## Data Availability

The High-Sensitivity Troponin in the Evaluation of Patients with Suspected Acute Coronary Syndrome trial makes use of several routine electronic health care data sources that are linked, deidentified and held in a Secure Data Environment by DataLoch (https://dataloch.org/), which is accessible by approved individuals who have undertaken the necessary governance training. Access to these data and those from the external validation datasets of Advantageous Predictors of Acute Coronary Syndromes Evaluation, Improved Assessment of Chest Pain Trial, 2-Hour Accelerated Diagnostic Protocol to Assess Patients with Chest Pain Symptoms Using Contemporary Troponins as the Only Biomarker, Emergency Department Assessment of Chest Pain Score, Signal Peptide in Acute Coronary Events and Use of Abbott High Sensitivity Troponin I Assay in Acute Coronary Syndromes cohorts from Switzerland, Spain, Poland, Czech Republic, Australia, New Zealand and the United States can be obtained by contacting the corresponding author.

## Code availability

The R code used to evaluate the Collaboration for the Diagnosis and Evaluation of Acute Coronary Syndrome (CoDE-ACS) models is available through GitHub (https://github.com/DimitriosDoudesis/CoDE-ACS-NatureMedicine), and we have created an evaluation tool in R-shiny to enable other researchers to run the CoDE-ACS models using individual patient-level data (https://decision-support.shinyapps.io/code-acs/). The datasets used to derive the CoDE-ACS models make use of several routine electronic health care data sources that are linked, deidentified and held in a Secure Data Environment by DataLoch (https://dataloch.org/). Researchers wishing the source data and models to conduct an evaluation of CoDE-ACS at scale should contact the corresponding author to arrange governance training, approvals and access to our Secure Data Environment.
