# Supplementary material for: Machine learning for diagnosis of myocardial infarction using cardiac troponin concentrations
Source: Nat Med. 2023 May 11;29(5):1201–10. doi: 10.1038/s41591-023-02325-4 (PMC10202804; doi:10.1038/s41591-023-02325-4)

nature medicine

Article

https://doi.org/10.1038/s41591-023-02325-4

Machine learning for diagnosis of myocardial infarction using cardiac troponin concentrations

In the format provided by the authors and unedited

List of Tables

Supplementary Table 1. Diagnostic performance of guideline-recommended cardiac troponin thresholds for myocardial infarction and CoDE-ACS scores in the derivation cohort.............3

Supplementary Table 2. Diagnostic performance of statistical models in the derivation cohort. 4

Supplementary Table 3. Diagnostic performance of guideline-recommended cardiac troponin thresholds for myocardial infarction and CoDE-ACS score in the external validation cohort .5

Supplementary Table 4. Reclassification using CoDE-ACS scores compared to guideline recommended high-sensitivity cardiac troponin thresholds in the external validation cohort. The overall net reclassification improvement index was 0.22 (95%CI 0.20 to 0.24)...............6

Supplementary Table 5. Diagnostic performance of guideline-recommended cardiac troponin thresholds for myocardial infarction and CoDE-ACS score in a US validation cohort. 7

Supplementary Table 6. Diagnostic performance of the sex-specific 99th percentile combined with a change in cardiac troponin on serial measurement for myocardial infarction in the external validation cohort. A relative increase of 20% or 50% was applied where the presentation value was above or below the 99th percentile, respectively. 8

Supplementary Table 7. Diagnostic performance of CoDE-ACS in the external validation cohort using a composite endpoint of type 1, 4b, 4c or type 2 myocardial infarction..............9

Supplementary Table 8. Comparison of the effectiveness and diagnostic performance of the CoDE-ACS and 0/1-hour pathway at presentation and following serial measurements in 5,634 patients from the external validation cohort where serial testing was performed at 0 and 1 hour. 10

Supplementary Table 9. Comparison of the effectiveness and diagnostic performance of the CoDE-ACS and the high-sensitivity cardiac troponin HEART pathway at presentation and following serial measurements in 2,271 patients from the external validation cohort where serial testing was performed at 0 and 3 hours..11

Supplementary Table 10. Diagnostic performance of different CoDE-ACS scores at presentation in the external validation cohort...12

Supplementary Table 11. The hyper-parameter values used within the CoDE-ACS clinical decision-support tool..13

List of Figures

Extended Data Fig 1. Flow diagram illustrating the populations used to train CoDE-ACS models in patients with and without myocardial injury [1]Lancet. 2015 Dec 19;386(10012):2481-8 [2]Lancet. 2018 Sep 15;392(10151):919-928. 14

Extended Data Fig 2. Negative predictive value of the 5 ng/L risk stratification threshold at presentation in the derivation cohort across patient subgroups. Data are presented as a central estimate with 95% confidence intervals based on the Clopper-Pearson method....................15

Extended Data Fig 3. Importance permutation rank of the features in the XGBoost model...16

Extended Data Fig 4. Diagnostic performance of CoDE-ACS scores at presentation in the derivation cohort across patient subgroups. Data are presented as a central estimate with 95% confidence intervals based on the Clopper-Pearson method. 18

Extended Data Fig 5. Diagnostic performance of CoDE-ACS scores on serial troponin testing in the derivation cohort across patient subgroups. Data are presented as a central estimate with 95% confidence intervals based on the Clopper-Pearson method. 20

Extended Data Fig 6. Diagnostic performance of CoDE-ACS in the external validation cohort using serial troponin results. A) Receiver-operating-characteristic (ROC) curve illustrating discrimination of the CoDE-ACS for myocardial infarction. B) Calibration of the CoDE-ACS score with the observed proportion of patients with myocardial infarction. The dashed line represents perfect calibration. Each point represents 100 patients. Patients are grouped as low- (<3), intermediate- (3 to 60) or high-probability (≥61) of myocardial infarction. The darker shaded area represents the 95% confidence interval, while the ligther shaded area the 99% confidence interval. 22

Extended Data Fig 7. Diagnostic performance of CoDE-ACS scores on serial troponin testing in the external validation cohort across patient subgroups. Data are presented as a central estimate with 95% confidence intervals based on the Clopper-Pearson method....................23

Extended Data Fig 8. External validation of the performance of the CoDE-ACS pathway in 3,629 women (A) and 6,657 men (B) with possible myocardial infarction. 25

Extended Data Fig 9. Diagnostic performance of the CoDE-ACS score in the external validation cohorts by region (Europe, Australia, New Zealand and United States). Receiver-operating-characteristic (ROC) curve illustrating discrimination of the CoDE-ACS for myocardial infarction. 27

Extended Data Fig. 10 Diagnostic performance in 5,634 patients of the external validation cohort who had cardiac troponin measurements at presentation and 1 hour to enable (A) CoDE-ACS score to identify patients as low-probability of myocardial infarction and (B) the 0/1-hour pathway to rule out myocardial infarction at presentation in subgroups. Data are presented as a central estimate with 95% confidence intervals based on the Clopper-Pearson method..29

Supplementary Table 1. Diagnostic performance of guideline-recommended cardiac troponin thresholds for myocardial infarction and CoDE-ACS scores in the derivation cohort.

A. Rule-out threshold in patients without myocardial injury at presentation

	True negative	False negative	True positive	False positive	NPV (95% CI)	Sensitivity (95% CI)	Proportion ruled out
**Cardiac troponin threshold**							
<5 ng/L	2286	9	123	1381	99.6 (99.3-99.8)	93.2 (88.8-97.2)	60%
**CoDE-ACS score less than 3**							
Presentation	2810	13	119	857	99.5 (99.3-99.8)	90.2 (84.7-95.0)	74%
Serial testing	1291	6	126	168	99.5 (99.2-99.8)	95.5 (92.0-98.5)	82%

B. Diagnostic threshold in patients with myocardial injury at presentation

	True negative	False negative	True positive	False positive	PPV (95% CI)	Specificity (95% CI)	Proportion ruled in
**Cardiac troponin threshold**							
Sex-specific >99th percentile	0	0	3085	3154	49.4 (48.2-50.7)	-	100%
**CoDE-ACS score 61 or more**							
Presentation	2631	981	2104	523	80.1 (78.5-81.6)	83.4 (82.1-84.7)	42%
Serial testing	1825	832	2300	487	82.5 (81.1-83.9)	80.1 (78.4-81.6)	51%

Supplementary Table 2. Diagnostic performance of statistical models in the derivation cohort.

A. Model performance in patients without myocardial injury

	NPV (95% CI)	Sensitivity (95% CI)	Proportion ruled out	AUC	Brier score
**Presentation cardiac troponin**					
Logistic regression	99.5 (99.1-99.7)	90.7 (84.1-95.7)	67%	0.898 (0.874-0.923)	0.028
Naïve Bayes	99.5 (99.2-99.8)	90.1 (84.7-95.2)	65%	0.875 (0.850-0.900)	0.054
Random Forest	99.5 (99.2-99.7)	90.1 (84.8-95.3)	72%	0.904 (0.881-0.927)	0.028
XGBoost	99.5 (99.3-99.8)	90.2 (84.7-95.0)	74%	0.912 (0.892-0.932)	0.028
**Serial cardiac troponin**					
Logistic regression	99.6 (98.6-100.0)	99.2 (97.4-100.0)	51%	0.890 (0.860-0.921)	0.052
Naïve Bayes	98.0 (97.1-98.8)	83.5 (76.6-89.4)	68%	0.870 (0.834-0.906)	0.062
Random Forest	99.6 (99.3-99.9)	96.3 (92.8-99.2)	79%	0.964 (0.947-0.981)	0.047
XGBoost	99.5 (99.2-99.8)	95.5 (92.0-98.5)	82%	0.970 (0.955-0.985)	0.030

B. Model performance in patients with myocardial injury

	PPV (95% CI)	Specificity (95% CI)	Proportion ruled in	AUC	Brier score
**Presentation cardiac troponin**					
Logistic regression	80.0 (78.3-81.6)	84.2 (82.9-85.5)	40%	0.845 (0.836-0.855)	0.160
Naïve Bayes	80.1 (78.4-81.7)	84.9 (83.7-86.2)	38%	0.831 (0.821-0.841)	0.174
Random Forest	80.1 (78.6-81.6)	81.8 (80.3-83.2)	41%	0.869 (0.861-0.878)	0.148
XGBoost	80.1 (78.5-81.6)	83.4 (82.1-84.7)	42%	0.852 (0.842-0.861)	0.157
**Serial cardiac troponin**					
Logistic regression	82.8 (81.2-84.2)	80.1 (78.4-81.7)	49%	0.836 (0.825-0.847)	0.162
Naïve Bayes	82.2 (80.7-83.7)	80.1 (78.4-81.7)	47%	0.824 (0.813-0.836)	0.194
Random Forest	83.4 (82.0-84.8)	80.2 (78.5-81.8)	50%	0.855 (0.845-0.865)	0.163
XGBoost	82.5 (81.1-83.9)	80.1 (78.4-81.6)	51%	0.844 (0.834-0.855)	0.158

Supplementary Table 3. Diagnostic performance of guideline-recommended cardiac troponin thresholds for myocardial infarction and CoDE-ACS score in the external validation cohort

A. Low probability score

	True negative	False negative	True positive	False positive	NPV (95% CI)	Sensitivity (95% CI)	Proportion ruled out
**Cardiac troponin threshold**							
<5 ng/L	2811	8	451	1423	99.7 (99.5-99.8)	98.3 (97.8-98.6)	27%
**Low probability CoDE-ACS score less than 3**							
Presentation	6238	27	1272	2749	99.6 (99.4-99.7)	97.9 (97.6-98.2)	61%
Serial testing	7405	32	1267	1582	99.6 (99.4-99.7)	97.5 (97.2-97.8)	72%

B. High probability score

	True negative	False negative	True positive	False positive	PPV (95% CI)	Specificity (95% CI)	Proportion ruled in
**Cardiac troponin threshold**							
Sex-specific >99th percentile	8397	267	1032	590	63.6 (62.7-64.5)	93.4 (92.9-93.9)	16%
**High probability CoDE-ACS score 61 or more**							
Presentation	8729	505	794	258	75.5 (74.6-76.3)	97.1 (96.8-97.4)	10%
Serial testing	8544	200	1099	443	71.3 (70.4-72.1)	95.1 (94.6-95.5)	15%

Supplementary Table 4. Reclassification using CoDE-ACS scores compared to guideline recommended high-sensitivity cardiac troponin thresholds in the external validation cohort. The overall net reclassification improvement index was 0.22 (95%CI 0.20 to 0.24).

	**CoDE-ACS scores**			
**Cardiac troponin I thresholds**	*Low probability (<3)*	*Intermediate (3 to 60)*	*High probability (≥61)*	*% reclassified*
<5 ng/L	2817	2	0	0%
5 ng/L to 99th percentile	3445	2395	5	59%
>99th percentile	3	572	1047	35%

Supplementary Table 5. Diagnostic performance of guideline-recommended cardiac troponin thresholds for myocardial infarction and CoDE-ACS score in a US validation cohort.

A. Low probability score

	True negative	False negative	True positive	False positive	NPV (95% CI)	Sensitivity (95% CI)	Proportion ruled out
**Cardiac troponin threshold**							
<5 ng/L	139	0	64	1432	100 (98.6-100)	100 (98.6-100)	9%
**Low probability CoDE-ACS score less than 3**							
Presentation	772	1	63	735	99.9 (99.5-100)	98.4 (97.7-98.9)	49%
Serial testing	1073	3	61	434	99.7 (99.3-99.9)	95.3 (94.2-96.3)	68%

B. High probability score

	True negative	False negative	True positive	False positive	PPV (95% CI)	Specificity (95% CI)	Proportion ruled in
**Cardiac troponin threshold**							
Sex-specific >99th percentile	1258	19	45	249	15.3 (13.6-17.2)	83.5 (81.6-85.2)	19%
**High probability CoDE-ACS score 61 or more**							
Presentation	1492	40	24	15	61.5 (59.1-63.9)	99.0 (98.4-99.4)	2%
Serial testing	1483	27	37	24	60.7 (58.2-63.0)	98.4 (97.7-98.9)	4%

Supplementary Table 6. Diagnostic performance of the sex-specific 99th percentile combined with a change in cardiac troponin on serial measurement for myocardial infarction in the external validation cohort. A relative increase of 20% or 50% was applied where the presentation value was above or below the 99th percentile, respectively.

A. Rule-out

	True negative	False negative	True positive	False positive	NPV (95% CI)	Sensitivity (95% CI)	Proportion ruled out
Serial testing	8663	628	671	324	93.2 (92.7-95.0)	51.7 (50.7-52.6)	90%

B. Rule-in

	True negative	False negative	True positive	False positive	PPV (95% CI)	Specificity (95% CI)	Proportion ruled in
Serial testing	8663	628	671	324	67.4 (66.5-68.3)	96.4 (96.0-96.7)	10%

Supplementary Table 7. Diagnostic performance of CoDE-ACS in the external validation cohort using a composite endpoint of type 1, 4b, 4c or type 2 myocardial infarction.

A. Low probability score

	True negative	False negative	True positive	False positive	NPV (95% CI)	Sensitivity (95% CI)	Proportion ruled out
**Low probability CoDE-ACS score less than 3**							
Presentation	6219	46	1553	2468	99.3 (99.1-99.4)	97.1 (96.8-97.4)	61%
Serial testing	7383	54	1545	1304	99.3 (99.1-99.4)	96.6 (96.3-97.0)	72%

B. High probability score

	True negative	False negative	True positive	False positive	PPV (95% CI)	Specificity (95% CI)	Proportion ruled in
**High probability CoDE-ACS score 61 or more**							
Presentation	8487	747	852	200	81.0 (80.2-81.7)	97.7 (97.4-98.0)	10%
Serial testing	8358	386	1213	329	78.7 (77.9-79.4)	96.2 (95.8-96.6)	15%

Supplementary Table 8. Comparison of the effectiveness and diagnostic performance of the CoDE-ACS and 0/1-hour pathway at presentation and following serial measurements in 5,634 patients from the external validation cohort where serial testing was performed at 0 and 1 hour.

A. Low probability score

	True negative	False negative	True positive	False positive	NPV (95% CI)	Sensitivity (95% CI)	Proportion ruled out
**0/1-hour pathway**							
Presentation	1505	1	773	3355	99.9 (99.8-100)	99.9 (99.7-99.9)	27%
Serial testing	2858	1	773	2002	100 (99.9-100)	99.9 (99.7-99.9)	51%
**Low probability CoDE-ACS score less than 3**							
Presentation	3201	9	765	1659	99.7 (99.5-99.8)	98.8 (98.5-99.1)	57%
Serial testing	3840	10	764	1020	99.7 (99.6-99.8)	98.7 (98.4-99.0)	63%

B. High probability score

	True negative	False negative	True positive	False positive	PPV (95% CI)	Specificity (95% CI)	Proportion ruled in
**0/1-hour pathway**							
Presentation	4637	286	488	223	68.6 (67.4-69.8)	95.4 (94.8-95.9)	13%
Serial testing	4443	86	688	417	62.3 (61.0-63.5)	91.4 (90.7-92.1)	20%
**High probability CoDE-ACS score 61 or more**							
Presentation	4669	269	505	191	72.6 (71.4-73.7)	96.1 (95.5-96.5)	12%
Serial testing	4538	96	678	322	67.8 (66.6-69.0)	93.4 (92.7-94.0)	18%

Supplementary Table 9. Comparison of the effectiveness and diagnostic performance of the CoDE-ACS and the high-sensitivity cardiac troponin HEART pathway at presentation and following serial measurements in 2,271 patients from the external validation cohort where serial testing was performed at 0 and 3 hours.

A. Low probability score

	True negative	False negative	True positive	False positive	NPV (95% CI)	Sensitivity (95% CI)	Proportion ruled out
**HEART pathway**							
Presentation	-	-	-	-	-	-	-
Serial testing	374	0	360	1537	100 (99.8-100)	100 (99.8-100)	16%
**Low probability CoDE-ACS score less than 3**							
Presentation	1164	5	355	747	99.6 (99.2-99.8)	98.6 (98.0-99.0)	51%
Serial testing	1504	5	355	407	99.7 (99.3-99.8)	98.6 (98.0-99.0)	66%

B. High probability score

	True negative	False negative	True positive	False positive	PPV (95% CI)	Specificity (95% CI)	Proportion ruled in
**HEART pathway**							
Presentation	-	-	-	-	-	-	-
Serial testing	374	0	360	1537	19.0 (17.4-20.6)	19.6 (18.0-21.3)	84%
**High probability CoDE-ACS score 61 or more**							
Presentation	1818	127	233	93	71.5 (69.6-73.3)	95.1 (94.2-95.9)	14%
Serial testing	1772	41	319	139	69.7 (67.7-71.5)	92.7 (91.6-93.7)	20%

Supplementary Table 10. Diagnostic performance of different CoDE-ACS scores at presentation in the external validation cohort.

	Threshold	True negative	False negative	True positive	False positive	NPV (95% CI)	Sensitivity (95% CI)	Proportion ruled out
**Low probability CoDE-ACS scores**								
More conservative	1	2470	2	1297	6517	99.9 (99.8-100.0)	99.8 (99.7-99.9)	24%
More conservative	2	5120	15	1284	3867	99.7 (99.6-99.8)	98.8 (98.6-99.0)	50%
**Selected**	**3**	**6238**	**27**	**1272**	**2749**	**99.6 (99.4-99.7)**	**97.9 (97.6-98.2)**	**61%**
Less conservative	4	6743	36	1263	2244	99.5 (99.3-99.6)	97.2 (96.9-97.5)	66%
Less conservative	5	7037	47	1252	1950	99.3 (99.2-99.5)	96.4 (96.0-96.7)	69%

		True negative	False negative	True positive	False positive	PPV (95% CI)	Specificity (95% CI)	Proportion ruled in
**High probability CoDE-ACS scores**								
Less conservative	50	8627	394	905	360	71.5 (70.7-72.4)	96.0 (95.6-96.4)	12%
Less conservative	55	8675	439	860	312	73.4 (72.5-74.2)	96.5 (96.2-96.9)	11%
**Selected**	**61**	**8729**	**505**	**794**	**258**	**75.5 (74.6-76.3)**	**97.1 (96.8-97.4)**	**10%**
More conservative	65	8751	551	748	236	76.0 (75.2-76.8)	97.4 (97.0-97.7)	10%
More conservative	70	8792	615	684	195	77.8 (77.0-78.6)	97.8 (97.5-98.1)	9%
More conservative	75	8833	686	613	154	80.0 (79.1-80.7)	98.3 (98.0-98.5)	7%

Supplementary Table 11. The hyper-parameter values used within the CoDE-ACS clinical decision-support tool.

	**Presentation cardiac troponin concentration**		**Serial cardiac troponin concentration**	
	**Without myocardial injury**	**With myocardial injury**	**Without myocardial injury**	**With myocardial injury**
Number of iterations	183	178	20	244
Learning rate	0.03	0.20	0.33	0.26
Interaction depth	8	2	4	1
Minimum number of observations in the terminal nodes	4	2	2	4
Fraction of the training set observations randomly selected for each subsequent tree	0.71	0.65	0.85	0.72
Fraction of variables randomly sampled for each tree	0.52	0.92	0.83	0.90

The algorithm was developed using the R package ‘xgboost’ version 1.6.2.

(https://cran.r-project.org/web/packages/xgboost/).

Extended Data Fig 1. Flow diagram illustrating the populations used to train CoDE-ACS models in patients with and without myocardial injury. [1]Lancet. 2015 Dec 19;386(10012):2481-8 [2]Lancet. 2018 Sep 15;392(10151):919-928

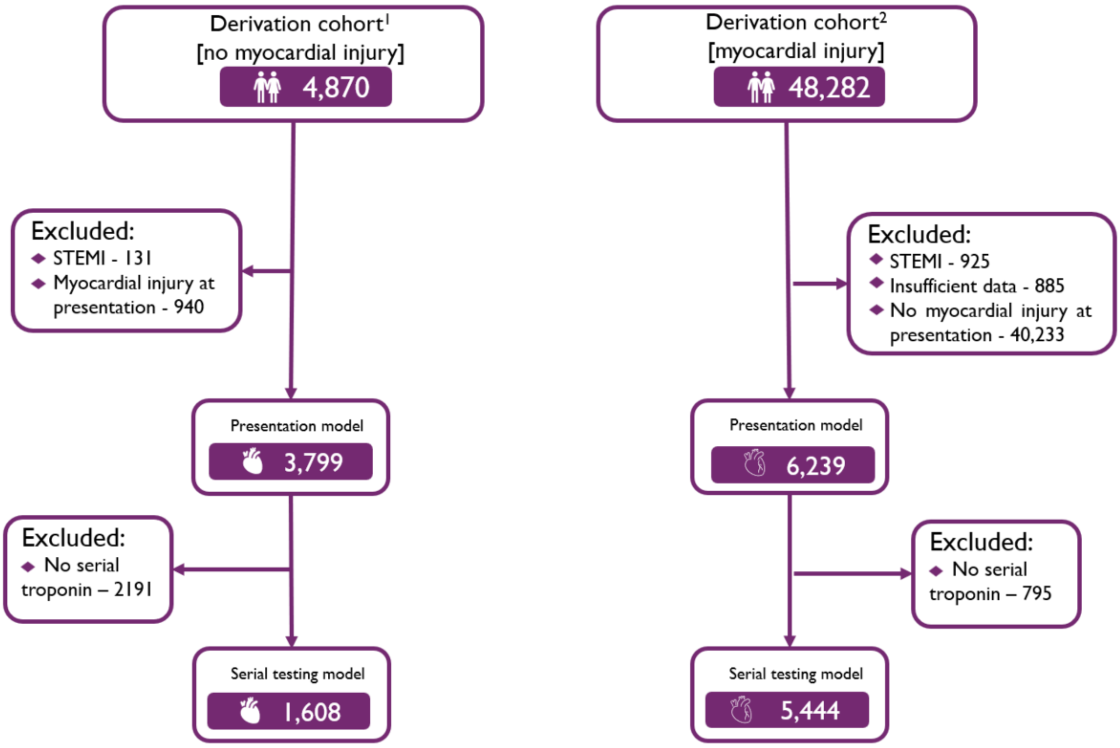

Extended Data Fig 2. Negative predictive value of the 5 ng/L risk stratification threshold at presentation in the derivation cohort across patient subgroups. Data are presented as a central estimate with 95% confidence intervals based on the Clopper-Pearson method.

Subgroups	True negative	False negative	Negative predictive value (95% CI)
**Age**			
<65 years	1592	5	99.7 (99.4-99.9)
≥65 years	694	4	99.4 (98.8-99.9)
**Sex**			
Male	1221	4	99.7 (99.3-99.9)
Female	1065	5	99.5 (99.1-99.9)
**Ischemic heart disease**			
Yes	481	6	98.7 (97.7-99.6)
No	1489	3	99.8 (99.5-100.0)
**Cerebrovascular disease**			
Yes	103	0	100.0 (100.0-100.0)
No	1863	9	99.5 (99.2-99.8)
**Diabetes**			
Yes	250	1	99.6 (98.7-100.0)
No	1717	8	99.5 (99.2-99.8)
**eGFR**			
<60 ml/min	153	2	98.7 (96.6-100.0)
≥60 ml/min	1757	7	99.6 (99.3-99.9)
**Ischemic electrocardiogram**			
Yes	179	3	98.3 (96.2-100.0)
No	1815	5	99.7 (99.5-99.9)
**Time from onset of symptoms**			
≤3 hours	563	8	98.6 (97.5-99.5)
>3 hours	1723	1	99.9 (99.8-100.0)
**Overall**	**2286**	**9**	**99.6 (99.3-99.8)**

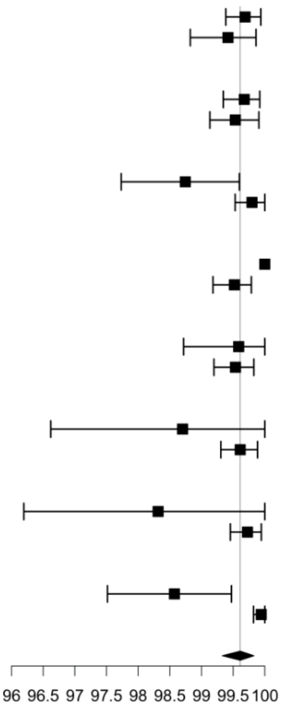

Extended Data Fig 3. Importance permutation rank of the features in the XGBoost model.

A) In patients without myocardial injury.

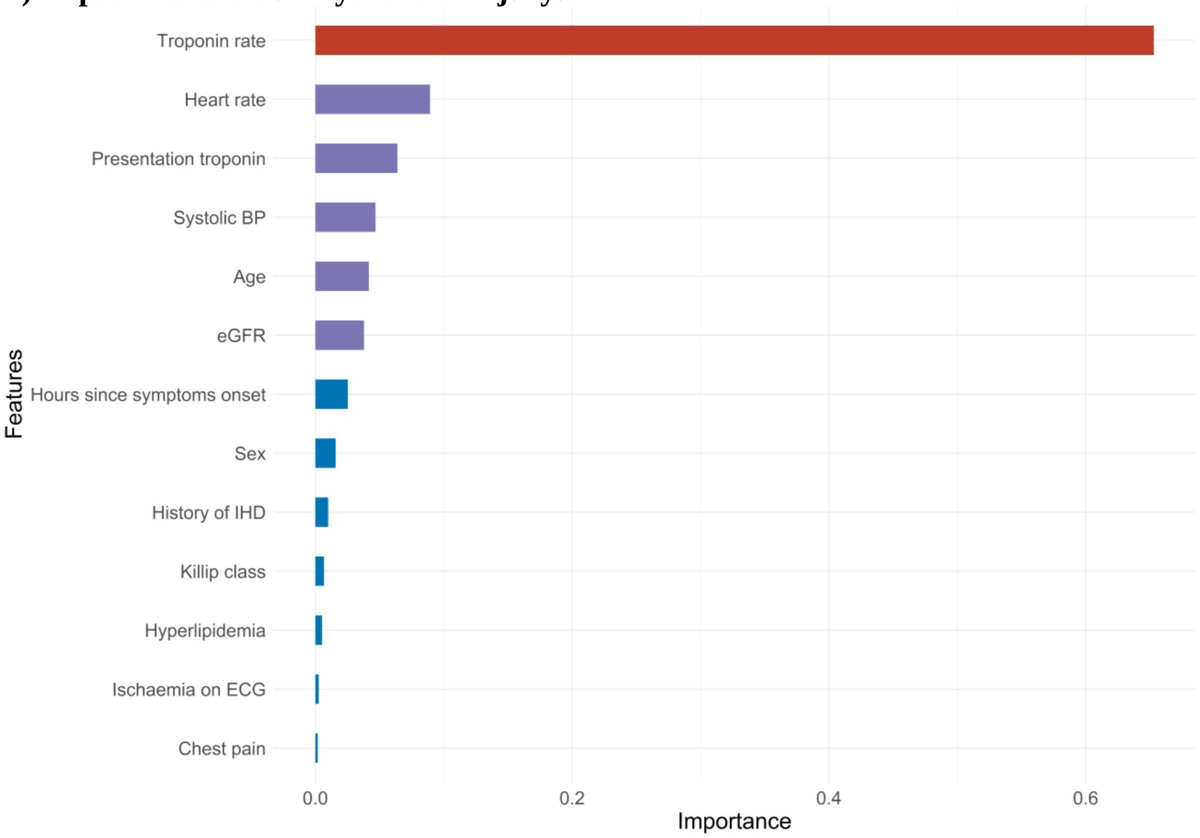

B) In patients with myocardial injury.

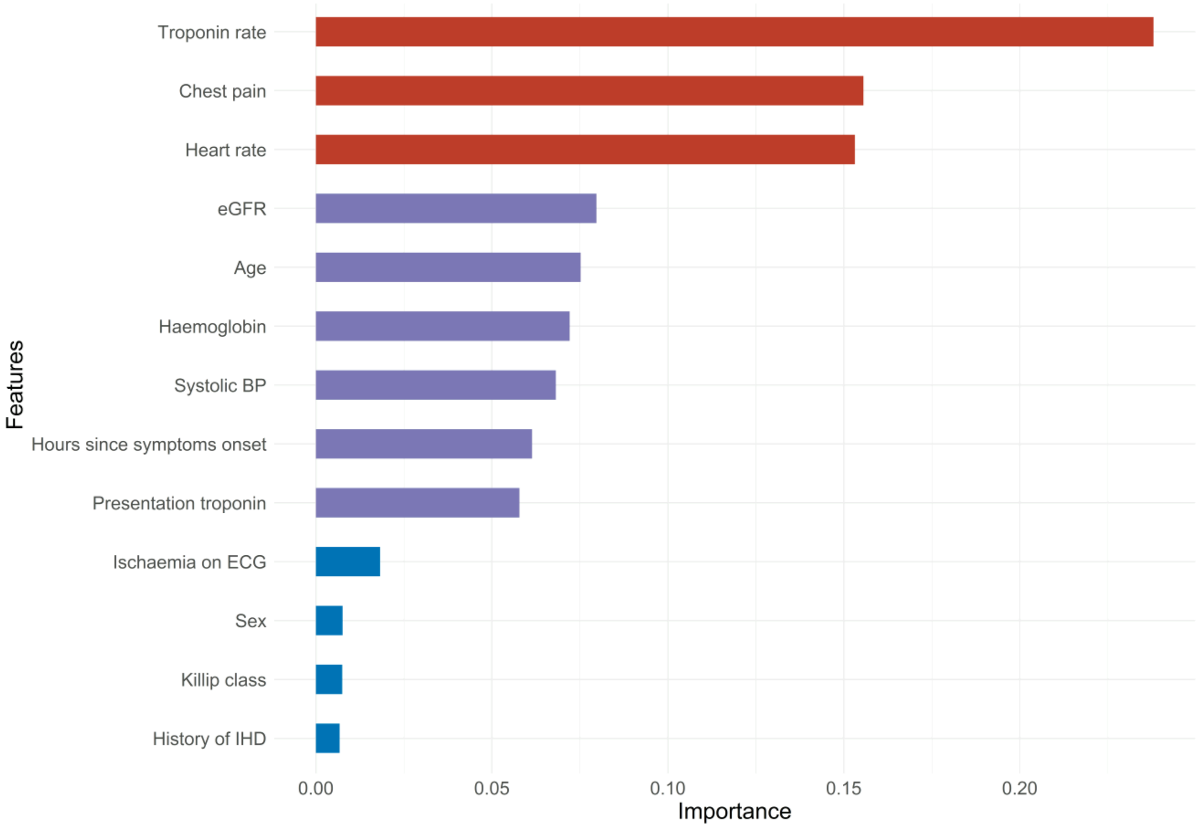

Extended Data Fig 4. Diagnostic performance of CoDE-ACS scores at presentation in the derivation cohort across patient subgroups. Data are presented as a central estimate with 95% confidence intervals based on the Clopper-Pearson method.

A) CoDE-ACS low probability score of less than 3

Subgroups	True negative	False negative	Negative predictive value (95% CI)
**Age**			
<65 years	1794	4	99.8 (99.5-99.9)
≥65 years	1016	9	99.1 (98.5-99.6)
**Sex**			
Male	1551	4	99.7 (99.5-99.9)
Female	1259	9	99.3 (98.8-99.7)
**Ischemic heart disease**			
Yes	622	8	98.7 (97.8-99.5)
No	1769	5	99.7 (99.4-99.9)
**Cerebrovascular disease**			
Yes	139	0	100.0 (100.0-100.0)
No	2244	13	99.4 (99.1-99.7)
**Diabetes**			
Yes	306	1	99.7 (99.0-100.0)
No	2080	12	99.4 (99.1-99.7)
**eGFR**			
<60 ml/min	211	3	98.6 (96.8-100.0)
≥60 ml/min	2095	9	99.6 (99.3-99.8)
**Ischemic electrocardiogram**			
Yes	179	1	99.4 (98.2-100.0)
No	2217	10	99.6 (99.3-99.8)
**Time from onset of symptoms**			
≤3 hours	513	6	98.8 (97.8-99.6)
>3 hours	2297	7	99.7 (99.4-99.9)
**Overall**	**2810**	**13**	**99.5 (99.3-99.8)**

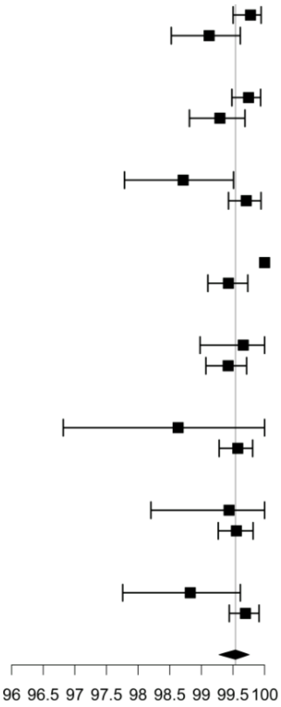

B) CoDE-ACS high probability score of 61 or more

Subgroups	True positive	False positive	Positive predictive value (95% CI)
**Age**			
<65 years	1018	200	83.6 (81.5-85.6)
≥65 years	1086	323	77.1 (74.9-79.4)
**Sex**			
Male	1367	274	83.3 (81.7-85.1)
Female	737	249	74.8 (71.9-77.4)
**Ischemic heart disease**			
Yes	553	221	71.4 (68.2-74.5)
No	1551	302	83.7 (82.0-85.4)
**Cerebrovascular disease**			
Yes	128	47	73.2 (66.8-79.5)
No	1976	476	80.6 (79.1-82.2)
**Diabetes**			
Yes	332	60	84.7 (81.1-88.1)
No	1772	463	79.3 (77.7-81.0)
**eGFR**			
<60 ml/min	414	152	73.2 (69.7-76.7)
≥60 ml/min	1657	355	82.4 (80.6-84.0)
**Ischemic electrocardiogram**			
Yes	752	138	84.5 (82.2-86.9)
No	1079	298	78.3 (76.1-80.4)
**Time from onset of symptoms**			
≤3 hours	676	177	79.3 (76.6-82.2)
>3 hours	1274	305	80.7 (78.7-82.6)
**Overall**	**2104**	**523**	**80.1 (78.6-81.5)**

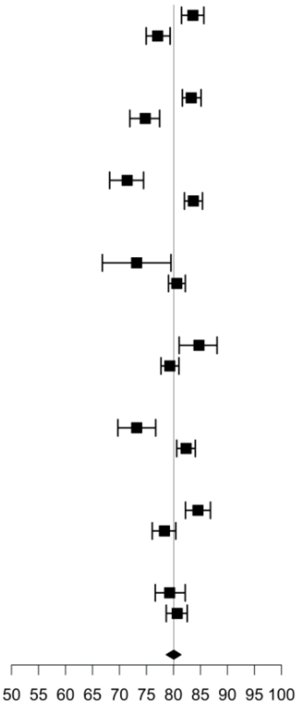

Extended Data Fig 5. Diagnostic performance of CoDE-ACS scores on serial troponin testing in the derivation cohort across patient subgroups. Data are presented as a central estimate with 95% confidence intervals based on the Clopper-Pearson method.

A) CoDE-ACS low probability score of less than 3

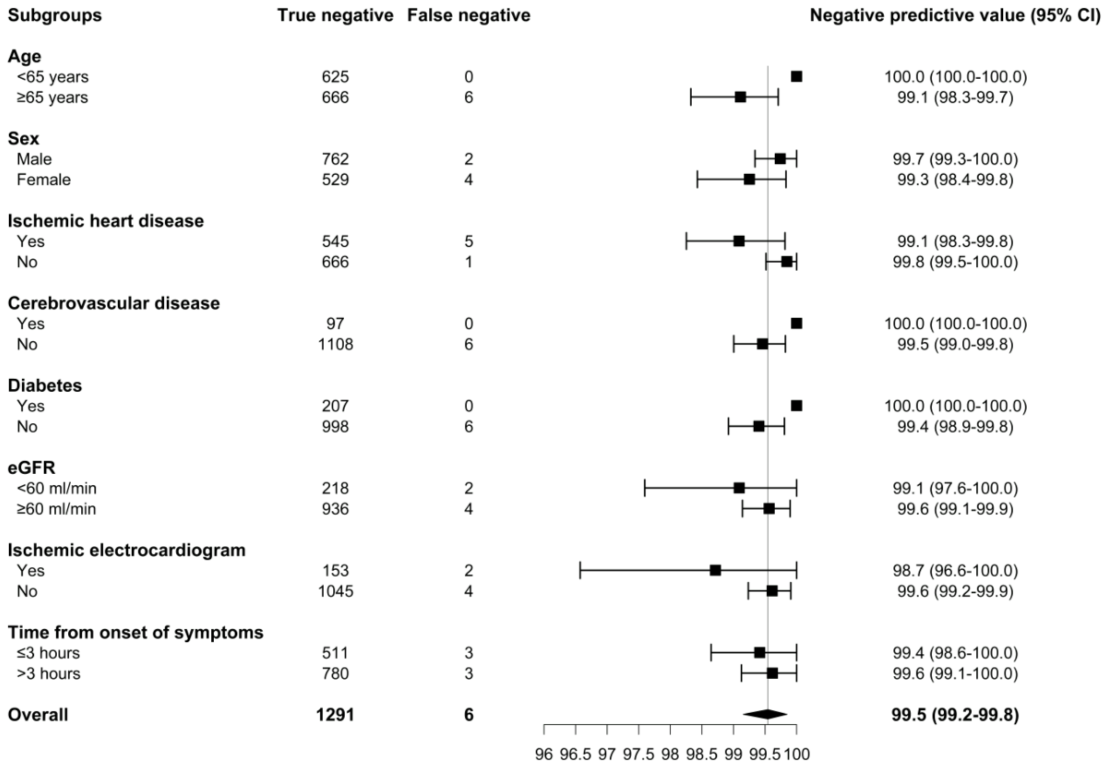

B) CoDE-ACS high probability score of 61 or more

Subgroups	True positive	False positive	Positive predictive value (95% CI)
**Age**			
<65 years	1073	191	84.9 (83.0-87.0)
≥65 years	1227	296	80.5 (78.5-82.5)
**Sex**			
Male	1487	256	85.3 (83.6-87.0)
Female	813	231	77.9 (75.2-80.5)
**Ischemic heart disease**			
Yes	754	202	78.9 (76.3-81.3)
No	1546	285	84.4 (82.7-86.1)
**Cerebrovascular disease**			
Yes	146	47	75.8 (69.7-81.9)
No	2154	440	83.1 (81.7-84.4)
**Diabetes**			
Yes	394	65	85.8 (82.6-88.7)
No	1906	422	81.9 (80.4-83.4)
**eGFR**			
<60 ml/min	490	130	79.0 (75.8-82.1)
≥60 ml/min	1769	345	83.7 (82.1-85.3)
**Ischemic electrocardiogram**			
Yes	761	129	85.5 (83.3-87.7)
No	1287	299	81.1 (79.2-83.1)
**Time from onset of symptoms**			
≤3 hours	937	191	83.1 (80.9-85.1)
>3 hours	1214	259	82.4 (80.5-84.3)
**Overall**	**2300**	**487**	**82.5 (81.2-84.0)**

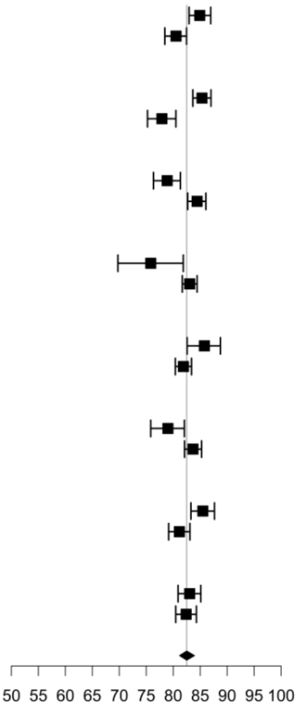

Extended Data Fig 6. Diagnostic performance of CoDE-ACS in the external validation cohort using serial troponin results.
A) Receiver-operating-characteristic (ROC) curve illustrating discrimination of the CoDE-ACS for myocardial infarction.
B) Calibration of the CoDE-ACS score with the observed proportion of patients with myocardial infarction. The dashed line represents perfect calibration. Each point represents 100 patients. Patients are grouped as low- (<3), intermediate- (3 to 60) or high-probability (≥61) of myocardial infarction. The darker shaded area represents the 95% confidence interval, while the ligther shaded area the 99% confidence interval.

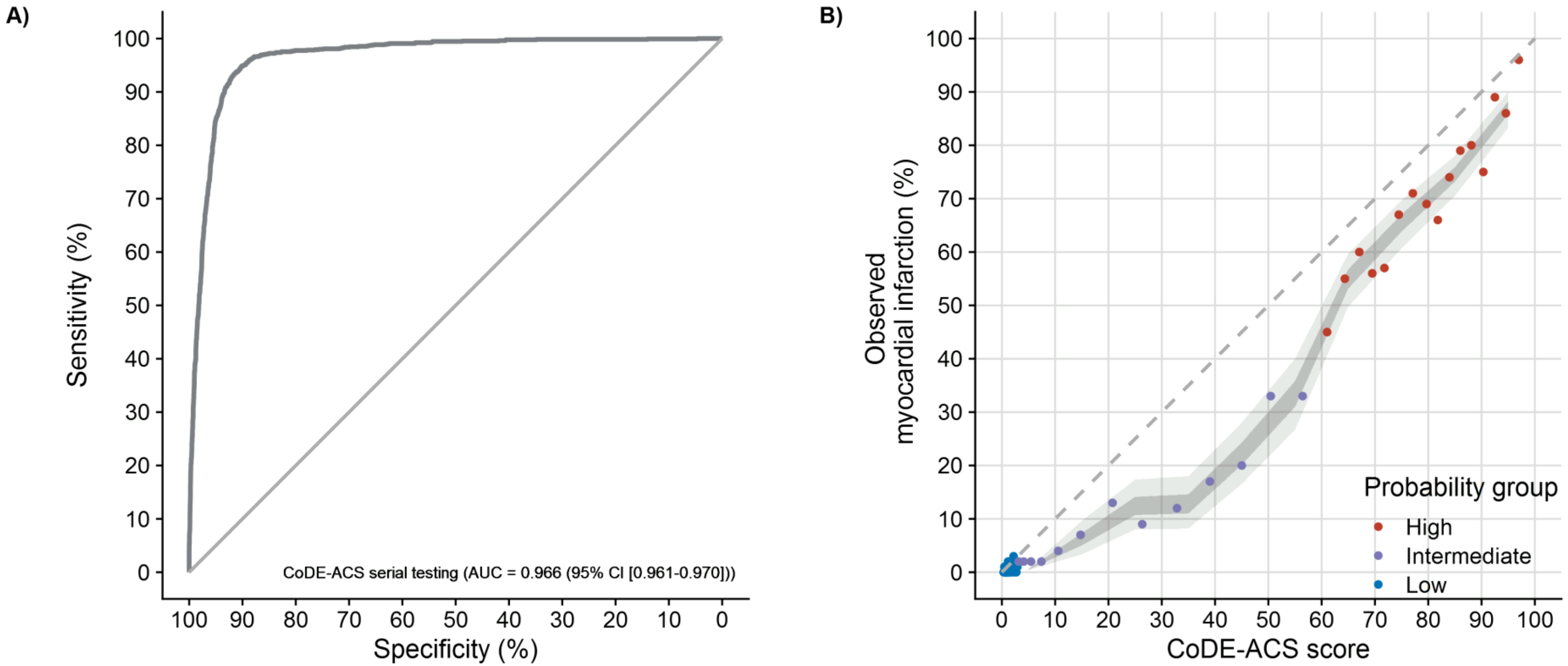

Extended Data Fig 7. Diagnostic performance of CoDE-ACS scores on serial troponin testing in the external validation cohort across patient subgroups. Data are presented as a central estimate with 95% confidence intervals based on the Clopper-Pearson method.

A) CoDE-ACS low probability score of less than 3

Subgroups	True negative	False negative	Negative predictive value (95% CI)	Proportion ruled out
**Age**				
<65 years	5365	12	99.8 (99.6-99.9)	84% [5377/6387]
≥65 years	2040	20	99.0 (98.7-99.3)	53% [2060/3899]
**Sex**				
Male	4709	25	99.5 (99.3-99.6)	71% [4734/6657]
Female	2696	7	99.7 (99.5-99.9)	74% [2703/3629]
**Ischemic heart disease**				
Yes	1807	13	99.3 (98.9-99.5)	59% [1820/3104]
No	5597	19	99.7 (99.5-99.8)	78% [5616/7181]
**Cerebrovascular disease**				
Yes	317	1	99.7 (98.8-99.9)	55% [318/582]
No	7088	31	99.6 (99.4-99.7)	73% [7119/9704]
**Diabetes**				
Yes	958	8	99.2 (98.6-99.5)	59% [966/1643]
No	6441	24	99.6 (99.5-99.7)	75% [6465/8635]
**eGFR**				
<60 ml/min	654	8	98.8 (98.1-99.2)	44% [662/1488]
≥60 ml/min	6358	21	99.7 (99.5-99.8)	78% [6379/8197]
**Ischemic electrocardiogram**				
Yes	503	2	99.6 (99.1-99.8)	36% [505/1396]
No	6862	30	99.6 (99.4-99.7)	78% [6892/8823]
**Time from onset of symptoms**				
≤3 hours	3232	6	99.8 (99.6-99.9)	71% [3238/4535]
>3 hours	4044	26	99.4 (99.1-99.5)	73% [4070/5572]
**Overall**	**7405**	**32**	**99.6 (99.4-99.7)**	**72% [7437/10286]**

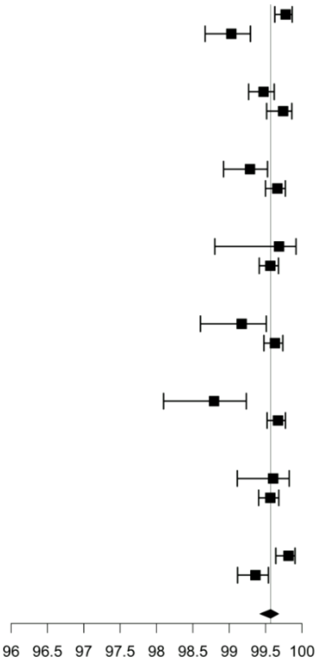

B) CoDE-ACS high probability score of 61 or more

Subgroups	True positive	False positive	Positive predictive value (95% CI)	Proportion ruled in
**Age**				
<65 years	464	203	69.6 (68.4-70.7)	10% [667/6387]
≥65 years	635	240	72.6 (71.1-73.9)	22% [875/3899]
**Sex**				
Male	826	274	75.1 (74.0-76.1)	17% [1100/6657]
Female	273	169	61.8 (60.2-63.3)	12% [442/3629]
**Ischemic heart disease**				
Yes	445	174	71.9 (70.3-73.4)	20% [619/3104]
No	654	269	70.9 (69.8-71.9)	13% [923/7181]
**Cerebrovascular disease**				
Yes	85	26	76.6 (73.0-79.8)	19% [111/582]
No	1014	417	70.9 (69.9-71.8)	15% [1431/9704]
**Diabetes**				
Yes	261	87	75.0 (72.8-77.0)	21% [348/1643]
No	838	355	70.2 (69.3-71.2)	14% [1193/8635]
**eGFR**				
<60 ml/min	236	98	70.7 (68.3-72.9)	22% [334/1488]
≥60 ml/min	796	326	70.9 (70.0-71.9)	14% [1122/8197]
**Ischemic electrocardiogram**				
Yes	448	137	76.6 (74.3-78.7)	42% [585/1396]
No	637	305	67.6 (66.6-68.6)	11% [942/8823]
**Time from onset of symptoms**				
≤3 hours	416	218	65.6 (64.2-67.0)	14% [634/4535]
>3 hours	671	220	75.3 (74.2-76.4)	16% [891/5572]
**Overall**	**1099**	**443**	**71.3 (70.4-72.1)**	**15% [1542/10286]**

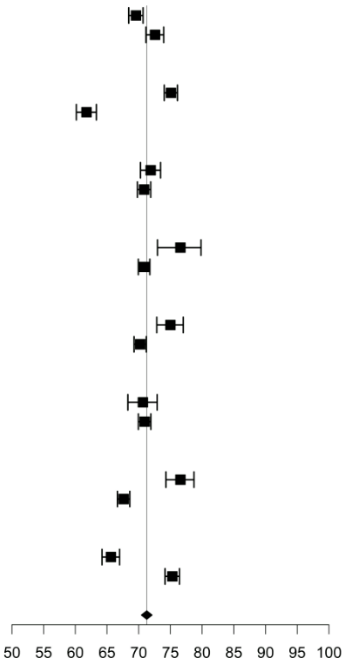

Extended Data Fig 8. External validation of the performance of the CoDE-ACS pathway in 3,629 women (A) and 6,657 men (B) with possible myocardial infarction.

A)

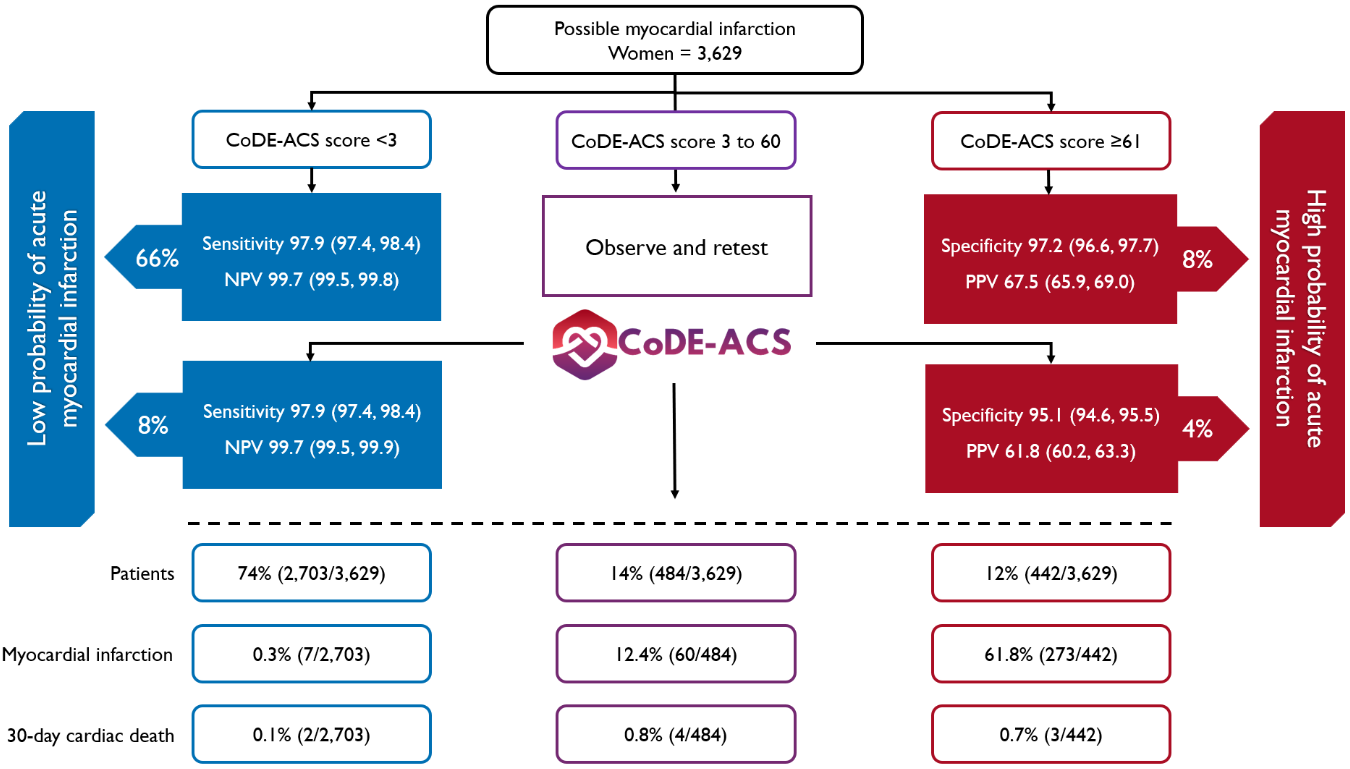

B)

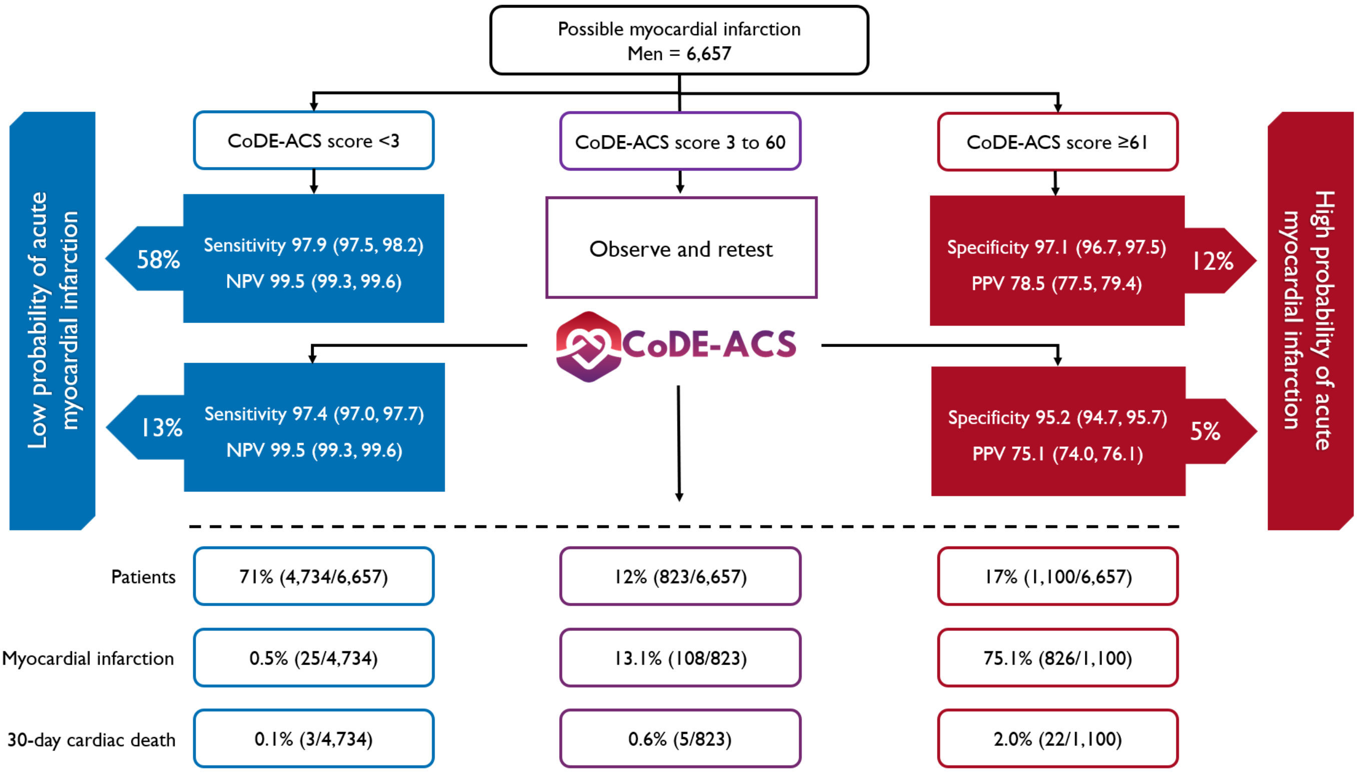

Possible myocardial infarction
Men = 6,657
CoDE-ACS score <3
CoDE-ACS score 3 to 60
CoDE-ACS score ≥61
Low probability of acute myocardial infarction
58%
Sensitivity 97.9 (97.5, 98.2)
NPV 99.5 (99.3, 99.6)
Observe and retest
Specificity 97.1 (96.7, 97.5)
PPV 78.5 (77.5, 79.4)
12%
High probability of acute myocardial infarction
CoDE-ACS
13%
Sensitivity 97.4 (97.0, 97.7)
NPV 99.5 (99.3, 99.6)
Specificity 95.2 (94.7, 95.7)
PPV 75.1 (74.0, 76.1)
5%
Patients
71% (4,734/6,657)
12% (823/6,657)
17% (1,100/6,657)
Myocardial infarction
0.5% (25/4,734)
13.1% (108/823)
75.1% (826/1,100)
30-day cardiac death
0.1% (3/4,734)
0.6% (5/823)
2.0% (22/1,100)

Extended Data Fig 9. Diagnostic performance of the CoDE-ACS score in the external validation cohorts by region (Europe, Australia, New Zealand and United States). Receiver-operating-characteristic (ROC) curve illustrating discrimination of the CoDE-ACS for myocardial infarction.

A) Using the presentation cardiac troponin measurement

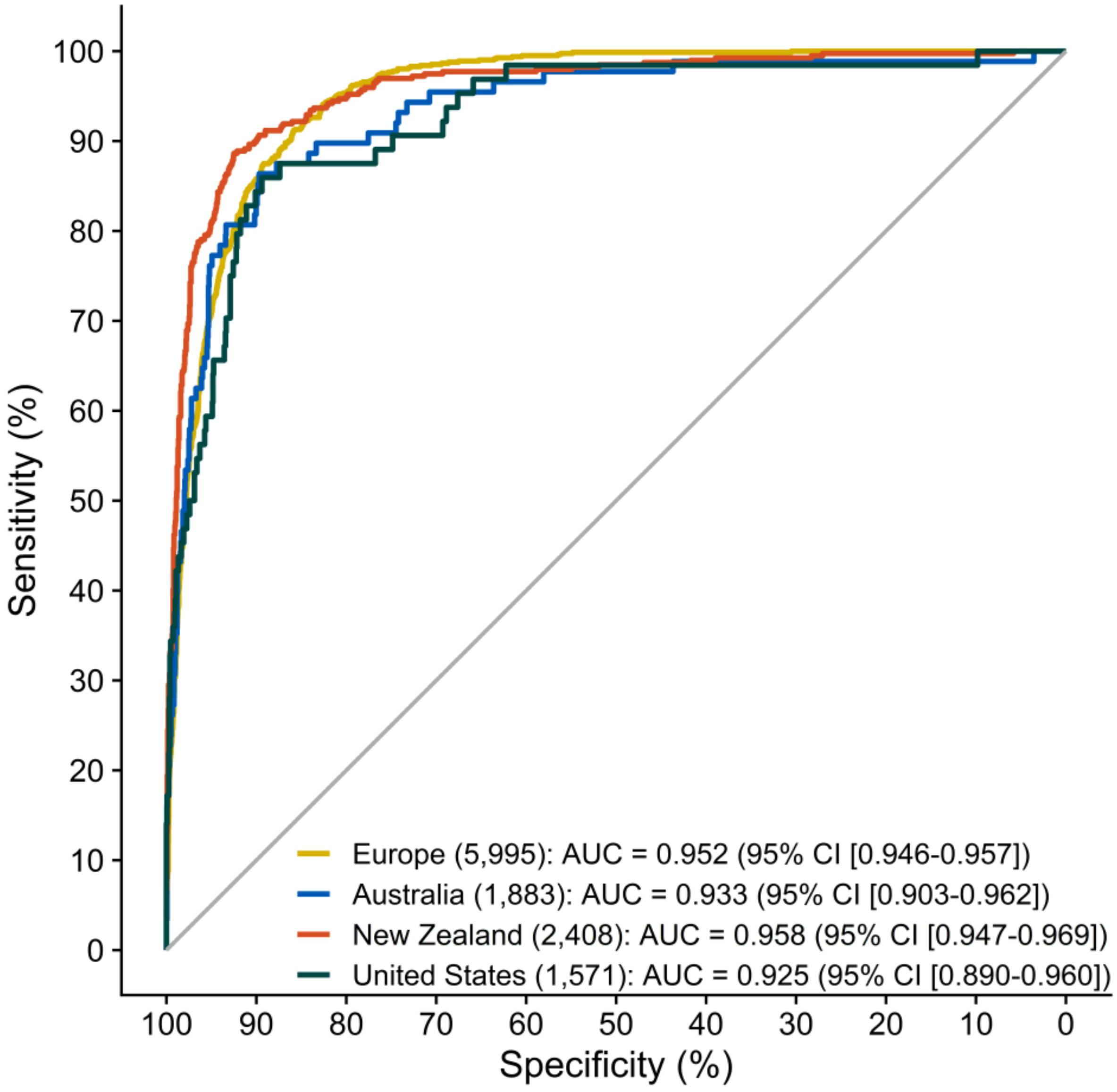

B) Using the serial cardiac troponin measurement

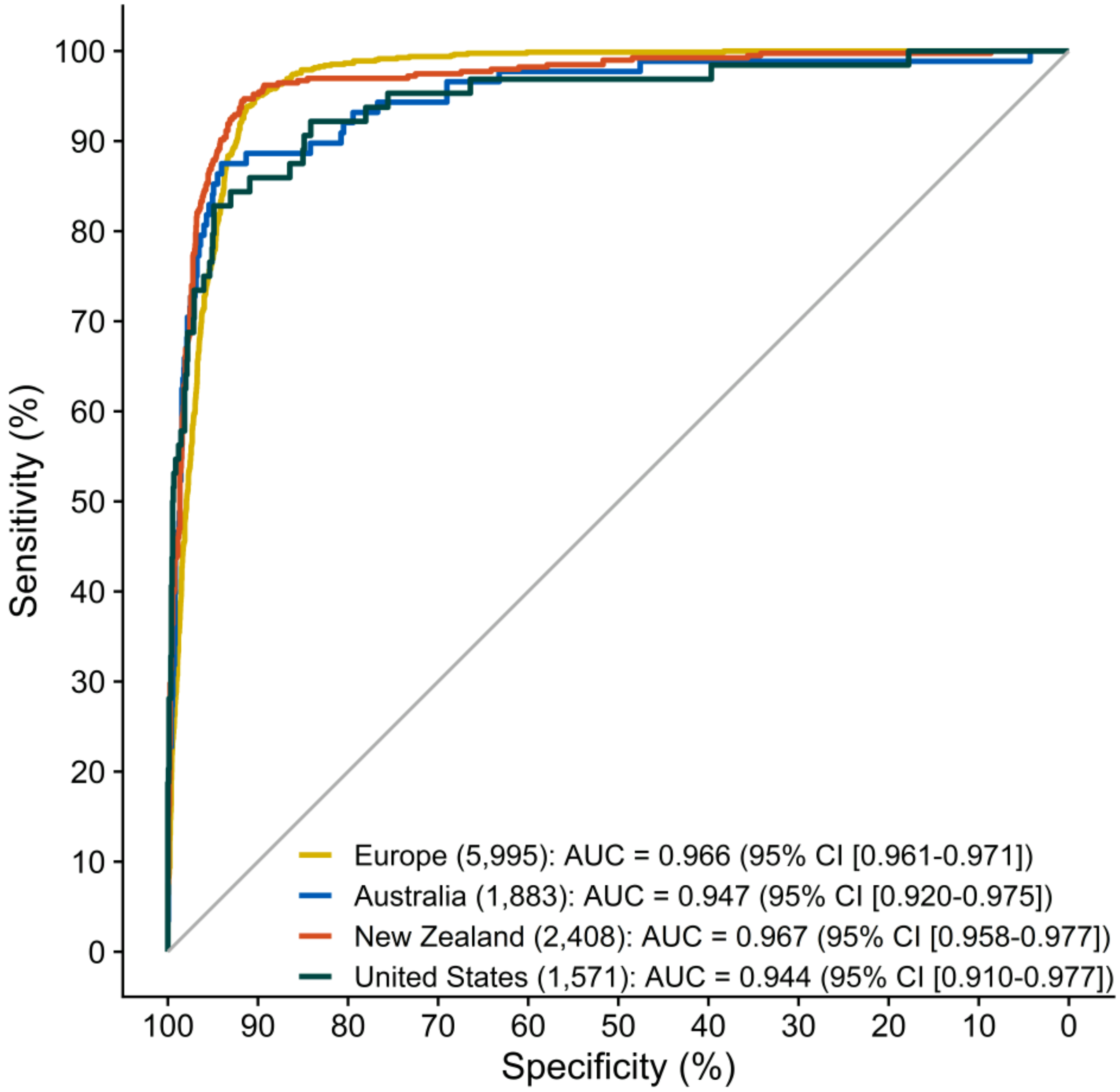

Extended Data Fig. 10 Diagnostic performance in 5,634 patients of the external validation cohort who had cardiac troponin measurements at presentation and 1 hour to enable (A) CoDE-ACS score to identify patients as low-probability of myocardial infarction and (B) the 0/1-hour pathway to rule out myocardial infarction at presentation in subgroups. Data are presented as a central estimate with 95% confidence intervals based on the Clopper-Pearson method.

A)

Subgroups	True negative	False negative	Negative predictive value (95% CI)	Proportion ruled out
**Age**				
<65 years	2358	2	99.9 (99.7-100.0)	72% [2360/3287]
≥65 years	843	7	99.2 (98.7-99.5)	36% [850/2347]
**Sex**				
Male	2068	6	99.7 (99.5-99.8)	54% [2074/3820]
Female	1133	3	99.7 (99.4-99.9)	63% [1136/1814]
**Ischemic heart disease**				
Yes	670	4	99.4 (98.9-99.7)	37% [674/1819]
No	2530	5	99.8 (99.6-99.9)	66% [2535/3814]
**Cerebrovascular disease**				
Yes	94	0	100.0 (98.7-100.0)	32% [94/290]
No	3107	9	99.7 (99.5-99.8)	58% [3116/5344]
**Diabetes**				
Yes	394	3	99.2 (98.5-99.6)	40% [397/988]
No	2804	6	99.8 (99.6-99.9)	61% [2810/4639]
**eGFR**				
<60 ml/min	206	2	99.0 (98.1-99.5)	25% [208/821]
≥60 ml/min	2896	6	99.8 (99.6-99.9)	63% [2902/4622]
**Ischemic electrocardiogram**				
Yes	161	0	100.0 (99.6-100.0)	16% [161/983]
No	3010	9	99.7 (99.5-99.8)	66% [3019/4594]
**Time from onset of symptoms**				
≤3 hours	1369	1	99.9 (99.7-100.0)	51% [1370/2709]
>3 hours	1789	8	99.6 (99.2-99.7)	63% [1797/2851]
**Overall**	**3201**	**9**	**99.7 (99.5-99.8)**	**57% [3210/5634]**

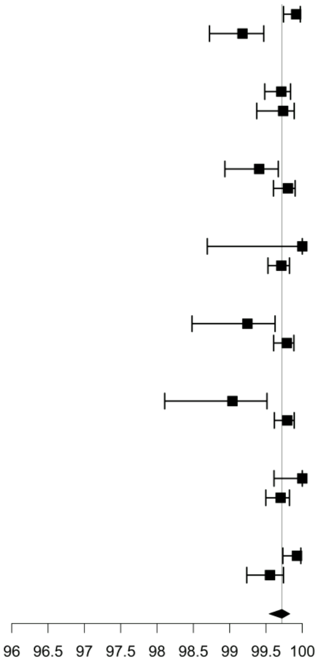

B)

Subgroups	True negative	False negative	Negative predictive value (95% CI)	Proportion ruled out
**Age**				
<65 years	1183	0	100.0 (99.9-100.0)	36% [1183/3287]
≥65 years	322	1	99.7 (99.4-99.8)	14% [323/2347]
**Sex**				
Male	933	1	99.9 (99.7-100.0)	24% [934/3820]
Female	572	0	100.0 (99.8-100.0)	32% [572/1814]
**Ischemic heart disease**				
Yes	287	1	99.7 (99.3-99.8)	16% [288/1819]
No	1217	0	100.0 (99.9-100.0)	32% [1217/3814]
**Cerebrovascular disease**				
Yes	29	0	100.0 (98.7-100.0)	10% [29/290]
No	1476	1	99.9 (99.8-100.0)	28% [1477/5344]
**Diabetes**				
Yes	164	1	99.4 (98.7-99.7)	17% [165/988]
No	1339	0	100.0 (99.9-100.0)	29% [1339/4639]
**eGFR**				
<60 ml/min	69	0	100.0 (99.5-100.0)	8% [69/821]
≥60 ml/min	1389	1	99.9 (99.8-100.0)	30% [1390/4622]
**Ischemic electrocardiogram**				
Yes	107	0	100.0 (99.6-100.0)	11% [107/983]
No	1386	1	99.9 (99.8-100.0)	30% [1387/4594]
**Time from onset of symptoms**				
≤3 hours	354	0	100.0 (99.9-100.0)	13% [354/2709]
>3 hours	1151	1	99.9 (99.7-100.0)	40% [1152/2851]
**Overall**	**1505**	**1**	**99.9 (99.8-100.0)**	**27% [1506/5634]**

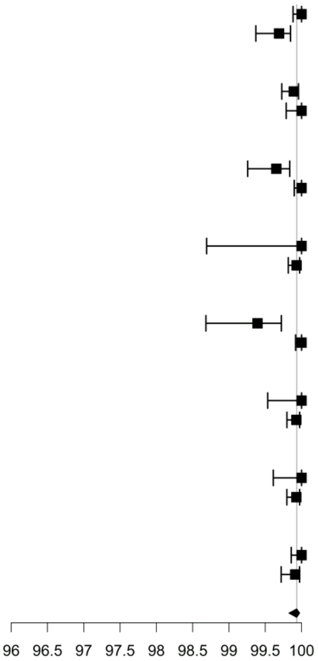

Supplement: Supplementary file 1 — Supplementary Figs. 1–10 and Tables 1–11. [file 41591_2023_2325_MOESM1_ESM.pdf]
