# Supplementary material for: Machine learning for diagnosis of myocardial infarction using cardiac troponin concentrations
Source: Nat Med. 2023 May 11;29(5):1201–10. doi: 10.1038/s41591-023-02325-4 (PMC10202804; doi:10.1038/s41591-023-02325-4)
Supplement: Supplementary file 2 — Reporting Summary [file 41591_2023_2325_MOESM2_ESM.pdf]

nature portfolio

Corresponding author(s): Professor Nicholas L. Mills

Last updated by author(s): Mar 21, 2023

Reporting Summary

Nature Portfolio wishes to improve the reproducibility of the work that we publish. This form provides structure for consistency and transparency in reporting. For further information on Nature Portfolio policies, see our Editorial Policies and the Editorial Policy Checklist.

Statistics

For all statistical analyses, confirm that the following items are present in the figure legend, table legend, main text, or Methods section.

n/a	Confirmed	
☐	☒	The exact sample size (*n*) for each experimental group/condition, given as a discrete number and unit of measurement
☐	☒	A statement on whether measurements were taken from distinct samples or whether the same sample was measured repeatedly
☐	☒	The statistical test(s) used AND whether they are one- or two-sided<br>*Only common tests should be described solely by name; describe more complex techniques in the Methods section.*
☐	☒	A description of all covariates tested
☐	☒	A description of any assumptions or corrections, such as tests of normality and adjustment for multiple comparisons
☐	☒	A full description of the statistical parameters including central tendency (e.g. means) or other basic estimates (e.g. regression coefficient) AND variation (e.g. standard deviation) or associated estimates of uncertainty (e.g. confidence intervals)
☐	☒	For null hypothesis testing, the test statistic (e.g. *F*, *t*, *r*) with confidence intervals, effect sizes, degrees of freedom and *P* value noted<br>*Give P values as exact values whenever suitable.*
☒	☐	For Bayesian analysis, information on the choice of priors and Markov chain Monte Carlo settings
☒	☐	For hierarchical and complex designs, identification of the appropriate level for tests and full reporting of outcomes
☒	☐	Estimates of effect sizes (e.g. Cohen's *d*, Pearson's *r*), indicating how they were calculated

Our web collection on statistics for biologists contains articles on many of the points above.

Software and code

Policy information about availability of computer code

Data collection	The High-STEACS trial makes use of several routine electronic health care data sources that are linked, de-identified, and held in a Secure Data Environment within the National Health Service by DataLoch (https://dataloch.org/), which is accessible by approved individuals who have undertaken the necessary governance training.
Data analysis	The R code used to evaluate the CoDE-ACS models is available through GitHub (https://github.com/DimitriosDoudesis/CoDE-ACS-NatureMedicine) and we have created an evaluation tool in R-shiny to enable other researchers to run the CoDE-ACS models using individual patient level data (https://decision-support.shinyapps.io/code-acs/). The datasets used to derive the CoDE-ACS models make use of several routine electronic health care data sources that are linked, de-identified, and held in a Secure Data Environment by DataLoch (https://dataloch.org/). Researchers wishing the source data and models to conduct an evaluation of CoDE-ACS at scale, should contact the corresponding author to arrange governance training, approvals, and access to our Secure Data Environment. All analyses were performed in R version 4.1.2. The algorithm was developed using the R package 'xgboost' version 1.6.2.

For manuscripts utilizing custom algorithms or software that are central to the research but not yet described in published literature, software must be made available to editors and reviewers. We strongly encourage code deposition in a community repository (e.g. GitHub). See the Nature Portfolio guidelines for submitting code & software for further information.

Data

Policy information about availability of data

All manuscripts must include a data availability statement. This statement should provide the following information, where applicable:

- Accession codes, unique identifiers, or web links for publicly available datasets
- A description of any restrictions on data availability
- For clinical datasets or third party data, please ensure that the statement adheres to our policy

The High-STEACS trial makes use of several routine electronic health care data sources that are linked, de-identified, and held in a Secure Data Environment by DataLoch (https://dataloch.org/), which is accessible by approved individuals who have undertaken the necessary governance training. Researchers wishing to access these data should contact the corresponding author to arrange governance training, approvals, and remote access to the Secure Data Environment. The external validation datasets of APACE (Advantageous Predictors of Acute Coronary Syndromes Evaluation), IMPACT (Improved Assessment of Chest pain Trial), ADAPT (2-Hour Accelerated Diagnostic Protocol to Assess Patients With Chest Pain Symptoms Using Contemporary Troponins as the Only Biomarker), EDACS (Emergency Department Assessment of Chest pain Score), SPACE (Signal Peptide in Acute Coronary Events) and UTROPIA (Use of Abbott High Sensitivity Troponin I Assay In Acute Coronary Syndromes) cohorts from Switzerland, Spain, Poland, Czech Republic, Australia, New Zealand and the United States can be accessed by contacting each corresponding author.

Human research participants

Policy information about studies involving human research participants and Sex and Gender in Research.

Reporting on sex and gender	Our manuscript is compliant with the journal's policy on sex and gender reporting. Sex was carefully considered in our study design. We previously demonstrated that cardiac troponin concentrations differ between men and women (BMJ. 2015;350:g7873), which informed the current international definition of myocardial infarction (Circulation. 2018;138:e618-e651). Sex is self-reported by patients when they register with the National Health Service, and this was used in the derivation cohorts for our models. In our external validation cohorts, sex was assigned by the researchers. Our models include sex as one of the variables used to estimate the probability of myocardial infarction. We reported a priori the overall performance of our model by sex. However, in our revised manuscript we have also included an additional post hoc evaluation of the diagnostic performance of the CoDE-ACS pathway separately in men and women and have discussed these findings.
Population characteristics	Patients were included in this prespecified secondary analysis based on the following criteria: (1) age ≥18 years old, (2) presentation with suspected acute coronary syndrome, (3) cardiac troponin measured using the ARCHITECTSTAT high-sensitivity cardiac troponin I assay (Abbott Laboratories), (4) availability of electrocardiographic and physiological data for diagnostic adjudication. Patients with a diagnosis of ST-segment elevation myocardial infarction were excluded given they undergo coronary revascularisation directly without troponin testing in the Emergency Department.
Recruitment	The High-STEACS trial population was used for the derivation of the CoDE-ACS (Collaboration for the Diagnosis and Evaluation of Acute Coronary Syndrome) algorithm. As previously described, High-STEACS was a stepped-wedged cluster-randomized controlled trial to evaluate the implementation of a high-sensitivity cardiac troponin I assay in consecutive patients with suspected acute coronary syndrome presenting to ten secondary and tertiary hospitals in Scotland between June 10, 2013, and March 3, 2016. As all consecutive patients were enrolled there is no selection bias in this trial.
Ethics oversight	The High-Sensitivity Troponin in the Evaluation of Patients With Suspected Acute Coronary Syndrome (High-STEACS) trial was registered (www.clinicaltrials.gov NCT01852123) and approved by the Scotland A Research Ethics Committee, the Public Benefit and Privacy Panel for Health and Social Care, and by each National Health Service (NHS) Health Board. This analysis was prespecified in the trial protocol and was performed according to a separate Statistical Analysis Plan. As the trial intervention was implemented at hospital level, consent was not sought from individual patients. All data were collected prospectively from the electronic patient record, deidentified and linked to regional and national registries in a data repository within a National Health Service managed Secure Data Environment (DataLoch, Edinburgh, United Kingdom). All cohort studies contributing to the external validation were approved by their respective local research ethics committee or institutional review board with written informed consent from participants.

Note that full information on the approval of the study protocol must also be provided in the manuscript.

Field-specific reporting

Please select the one below that is the best fit for your research. If you are not sure, read the appropriate sections before making your selection.

☒ Life sciences ☐ Behavioural & social sciences ☐ Ecological, evolutionary & environmental sciences

For a reference copy of the document with all sections, see nature.com/documents/nr-reporting-summary-flat.pdf

Life sciences study design

All studies must disclose on these points even when the disclosure is negative.

Sample size	No prior sample size calculation have been performed. All the available data for this study has been used.
Data exclusions	Patients with a diagnosis of ST-segment elevation myocardial infarction were excluded given they undergo coronary revascularisation directly without troponin testing in the Emergency Department.
Replication	All the analysis is reproducible. A random seed has been used throughout the analysis to make sure that there are no small differences (decimal points) to the results.
Randomization	No randomized comparisons are reported in this analysis.
Blinding	The diagnosis of myocardial infarction and clinical outcomes were adjudicated by a panel of clinicians who were blinded to the trial phase and the CoDE-ACS score.

Reporting for specific materials, systems and methods

We require information from authors about some types of materials, experimental systems and methods used in many studies. Here, indicate whether each material, system or method listed is relevant to your study. If you are not sure if a list item applies to your research, read the appropriate section before selecting a response.

Materials & experimental systems

n/a	Involved in the study	
☒	☐	Antibodies
☒	☐	Eukaryotic cell lines
☒	☐	Palaeontology and archaeology
☒	☐	Animals and other organisms
☐	☒	Clinical data
☒	☐	Dual use research of concern

Methods

n/a	Involved in the study	
☒	☐	ChIP-seq
☒	☐	Flow cytometry
☒	☐	MRI-based neuroimaging

Clinical data

Policy information about clinical studies

All manuscripts should comply with the ICMJE guidelines for publication of clinical research and a completed CONSORT checklist must be included with all submissions.

Clinical trial registration	NCT01852123
Study protocol	Study protocol is available through the trial registration site (https://clinicaltrials.gov/ProvidedDocs/23/NCT01852123/Prot_000.pdf)
Data collection	All consecutive patients with suspected acute coronary syndrome presenting to ten secondary and tertiary hospitals in Scotland between June 10, 2013, and March 3, 2016.
Outcomes	The model was trained to identify patients with an adjudicated diagnosis of type 1, type 4b or type 4c myocardial infarction during the index hospital admission. All primary and secondary outcomes were prespecified in the trial protocol. The diagnosis of myocardial infarction was adjudicated according to the Fourth Universal Definition of Myocardial Infarction by two clinicians independently, with a third reviewer providing consensus if there was disagreement. Regional and national registries were used to follow-up the trial population for one year. The cause of death was adjudicated by investigators masked to troponin concentrations during the index presentation.
