# Supplementary material for: Machine learning for diagnosis of myocardial infarction using cardiac troponin concentrations
Source: Nat Med. 2023 May 11;29(5):1201–10. doi: 10.1038/s41591-023-02325-4 (PMC10202804; doi:10.1038/s41591-023-02325-4)
Supplement: Supplementary file 3 — Checklist for the supervised clinical ML study. [file 41591_2023_2325_MOESM3_ESM.pdf]

Checklist for supervised clinical ML study

Before paper submission			
**Study design (Part 1)**	**Completed: page number**		**Notes if not completed**
The clinical problem in which the model will be employed is clearly detailed in the paper.	X	p. 3	
The research question is clearly stated.	X	p. 3	
The characteristics of the cohorts (training and test sets) are detailed in the text.	X	p. 22-35 Table 1	
The cohorts (training and test sets) are shown to be representative of real-world clinical settings.	X	p. 22-35, Table 1	Training cohort consists of unselected consecutive patients presenting in the Emergency Department. Testing cohorts are a mixture of consented patients and consecutive patients presenting to a United States Emergency Department.
The state-of-the-art solution used as a baseline for comparison has been identified and detailed.	X	p. 6-9	We have now also compared our solution to the current guideline recommended 0 and 1-hour pathway from the European Society of Cardiology and the HEART pathway, in addition to the widely used cardiac troponin thresholds in clinical practice reported previously.
**Data and optimization (Parts 2, 3)**	**Completed: page number**		**Notes if not completed**
The origin of the data is described and the original format is detailed in the paper.	X	p. 22-23	Previous paper has been published with the detailed description and is being cited (ref 25)
Transformations of the data before it is applied to the proposed model are described.	☐		There were no transformations of the data prior to this project. Logistic regression was performed with the use of mfp (Multivariable Fractional Polynomials)
The independence between training and test sets has been proven in the paper.	X	p. 22-35	Different cohorts have been used and a detailed description of each has been provided in the on-line methods.
Details on the models that were evaluated and the code developed to select the best model are provided.	X	p. 24-26 and p. 35-37; Suppleme ntal Table 2 and 11	Details provided including the hyperparameters used in
Is the input data type structured or unstructured?	X Structured		☐ Unstructured
**Model performance (Part 4)**	**Completed: page number**		**Notes if not completed**
The primary metric selected to evaluate algorithm performance (eg: AUC, F-score, etc) including the justification for selection, has been clearly stated.	X	p. 25	
The primary metric selected to evaluate the clinical utility of the model (eg PPV, NNT, etc)	X	p. 25	

including the justification for selection, has been clearly stated.			
The performance comparison between baseline and proposed model is presented with the appropriate statistical significance.	X	p. 6-7	There was no formal statistical comparison between baseline and proposed model, but confidence intervals are provided for all diagnostic metrics
**Model Examination (Parts 5)**	**Completed: page number**		**Notes if not completed**
Examination Technique 1[a]	X	Extended Data Fig. 9	Diagnostic performance of the CoDE-ACS score in the external validation cohorts by region (Europe, Australia, New Zealand and United States)
Examination Technique 2[a]	☐		
A discussion of the relevance of the examination results with respect to model/algorithm performance is presented.	X	p. 11-12	
A discussion of the feasibility and significance of model interpretability at the case level if examination methods are uninterpretable is presented.	X	p. 11-12	
A discussion of the reliability and robustness of the model as the underlying data distribution shifts is included.	X	p.15-16	
Common examination approaches based on study type:<br> For studies involving exclusively structured data coefficients and sensitivity analysis are often appropriate<br>* For studies involving unstructured data in the domains of image analysis or NLP: saliency maps (or equivalents) and sensitivity analysis are often appropriate			
**Reproducibility (Part 6): choose appropriate tier of transparency**			**Notes**
Tier 1: complete sharing of the code		☐	
Tier 2: allow a third party to evaluate the code for accuracy/fairness; share the results of this evaluation		☐	
Tier 3: release of a virtual machine (binary) for running the code on new data without sharing its details		X	We have created an evaluation tool in R-shiny to enable other researchers to run the CoDE-ACS models using individual patient level data (https://decision-support.shinyapps.io/code-acs/). The datasets and code used to derive the CoDE-ACS models make use of several routine electronic health care data sources that are linked, de-identified, and held in a Secure Data Environment managed by DataLoch (https://dataloch.org/).<br><br>Researchers wishing the source data and models to conduct an evaluation of CoDE-ACS at scale, should contact the corresponding author to arrange governance training, approvals, and access to our Secure Data Environment.

Tier 4: no sharing	☐	

PPV: Positive Predictive Value

NNT: Numbers Needed to Treat

[a] Common examination approaches based on study type: for studies involving exclusively structured data, coefficients and sensitivity analysis are often appropriate; for studies involving unstructured data in the domains of image analysis or natural language processing, saliency maps (or equivalents) and sensitivity analyses are often appropriate. Select 2 from this list or chose an appropriate technique, document each technique used on the appropriate line above.
